# Comparing the Impacts of Testosterone and Exercise on Lean Body Mass, Strength and Aerobic Fitness in Aging Men

**DOI:** 10.1186/s40798-024-00703-x

**Published:** 2024-04-02

**Authors:** Daniel J. Green, Lauren C. Chasland, Bu B. Yeap, Louise H. Naylor

**Affiliations:** 1 School of Human Sciences (Exercise and Sport Science), The University of Western Australia, Perth, WA 6009 Australia; 2 Allied Health Department, Fiona Stanley Hospital, Perth, WA Australia; 3 Medical School, University of Western Australia, Perth, WA Australia; 4 Department of Endocrinology and Diabetes, Fiona Stanley Hospital, Perth, WA Australia

**Keywords:** Skeletal muscle, Function, Anabolic, Body composition, Oxygen uptake

## Abstract

**Background:**

Based on the largely untested premise that it is a restorative hormone that may reverse the detrimental impacts of aging, prescription of testosterone (T) has increased in recent decades despite no new clinical indications. It is apparent that middle-aged and older men with low-normal serum T levels are considering T supplementation as an anti-aging strategy. At the same time, there is evidence that physical activity (PA) is at historical lows in the Western world. In this review, we compare the impacts of T treatment aimed at achieving physiological T concentrations in middle-aged and older men, alongside the impacts of ecologically relevant forms of exercise training. The independent, and possible combined, effects of T and exercise therapy on physiological outcomes such as aerobic fitness, body composition and muscular strength are addressed.

**Main Body:**

Our findings suggest that both T treatment and exercise improve lean body mass in healthy older men. If improvement in lean body mass is the primary aim, then T treatment could be considered, and the combination of T and exercise may be more beneficial than either in isolation. In terms of muscle strength in older age, an exercise program is likely to be more beneficial than T treatment (where the dose is aimed at achieving physiological concentrations), and the addition of such T treatment does not provide further benefit beyond that of exercise alone. For aerobic fitness, T at doses aimed at achieving physiological concentrations has relatively modest impacts, particularly in comparison to exercise training, and there is limited evidence as to additive effects. Whilst higher doses of T, particularly by intramuscular injection, may have larger impacts on lean body mass and strength, this must be balanced against potential risks.

**Conclusion:**

Knowing the impacts of T treatment and exercise on variables such as body composition, strength and aerobic fitness extends our understanding of the relative benefits of physiological and pharmacological interventions in aging men. Our review suggests that T has impacts on strength, body composition and aerobic fitness outcomes that are dependent upon dose, route of administration, and formulation. T treatment aimed at achieving physiological T concentrations in middle-aged and older men can improve lean body mass, whilst exercise training enhances lean body mass, aerobic fitness and strength. Men who are physically able to exercise safely should be encouraged to do so, not only in terms of building lean body mass, strength and aerobic fitness, but for the myriad health benefits that exercise training confers.

**Supplementary Information:**

The online version contains supplementary material available at 10.1186/s40798-024-00703-x.

## Background

Testosterone (T) is the primary male sex hormone responsible for sexual development and virilization. It plays an important role in maintenance of bone and muscle, libido, and sperm production. As men grow older T concentrations can decline, with some studies reporting that T levels decrease at a rate of 1–2% per year from the third decade of life onwards [1, 2]. Ageing occurs continuously and defining youth, middle and older ages are societal as well as physiological constructs, with somewhat arbitrary thresholds. The UK Biobank focussed on adults aged 40–69 years [3], in keeping with the European Male Ageing Study of men aged 40–70 years [4]; whereas studies such as the Health In Men Study (HIMS) [5] and the Concord Health and Ageing in Men Project (CHAMP) [6] investigated T in men aged 70 years and above. Therefore, for the purposes of this review, we define young men as aged < 40 years, middle-aged men as aged 40–69 years, and older men as aged ≥ 70 years. Older men, on average, have lower T concentrations compared to younger men [5, 7, 8]. In 394 healthy men aged 71–87 years, the 2.5th and 97.5th percentiles for T measured using mass spectrometry were 6.4–25.6 nmol/L, compared to 10.4–30.1 nmol/L in reproductively normal men aged 21–35 years [5, 7]. Within any age stratum, there is considerable variation in T values between individual men [9]. Evidence of impaired testicular production of T is apparent after the age of 70 years [10, 11].

The age-related decline in T concentrations coincides with accumulation of medical comorbidities [12, 13], and with reductions in aerobic fitness and strength and unfavourable changes in body composition [14–16]. It is, however, important to note that T concentrations are higher in older men who engage in a healthy lifestyle, inclusive of regular exercise [17]. Men with organic disorders of the hypothalamus, pituitary or testes which impair the production of T present with symptoms and signs of androgen deficiency [18]. Such men typically have T concentrations lower than expected in healthy men of comparable age, and their symptoms and signs of androgen deficiency respond promptly to treatment with T. However, older men with T concentrations appropriate for their age but lower than expected for younger men, may exhibit non-specific symptoms such as tiredness and fatigue, in the absence of hypothalamic, pituitary or testicular disease.

In the USA, T prescriptions increased 11-fold between 2001 and 2011, largely in middle-aged to older men, in the absence of new medical indications [19]. Although prescriptions have subsequently decreased [20–22], it is nonetheless apparent that some middle-aged and older men with low-normal serum T levels are considering T supplementation as an anti-aging strategy [23]. At the same time, there is evidence that physical activity is at historical lows in the Western world [24]. There has never been a more inactive population than twenty-first century humans and some have suggested that, from an evolutionary perspective, this unprecedented decline in physical activity underlies the contemporary increase in chronic diseases [25]. Despite the fact that there is a mature evidence-base that exercise training can arrest some age-associated changes in body composition, strength and cardiovascular function in humans, increasing physical activity remains a population health challenge, and pharmacological strategies that emulate the impacts of exercise present an appealing alternative [26].

This review summarizes what is currently known about the impact of T treatment, exercise and their combination on body composition, strength and aerobic fitness. Although there is a body of literature concerning the effects of anabolic androgenic steroid (AAS) abuse, this review focuses on (physiological) T treatment, designed to increase T levels within the physiological range in middle and older aged men. The papers we review to provide the following summary sections are available as an online supplement. We also present the consolidated outcomes of the recent Testosterone and Exercise (TEX) randomised trial [27–29], a 2 × 2 factorial placebo-controlled trial of men aged 50–70 years with low-normal serum T levels who were randomized to directly compare the combined and independent impacts of T treatment (in the physiological range) and supervised center-based exercise.

## Main Text

### Lean Body Mass: The Effects of Testosterone, Exercise, and Their Combination

Building muscle mass is arguably a primary driver for off-label T use. Lean body mass decreases from middle age at a rate of approximately 1% per year [30], with its decline associated with decreased independence, reduced quality of life and, ultimately, frailty and an increased risk of mortality [31]. The anabolic effects of T are well established, with mechanisms related to increased muscle protein synthesis [32, 33], stimulated satellite cell replication [34] and inhibition of muscle protein degradation [35, 36]. Whilst illicit anabolic–androgenic steroid (AAS) use is beyond the scope of this review, the anabolic actions of T are relevant to middle-to-older aged men who may experience muscle loss as they age, with consequent detrimental effects on health [31]. Similarly, exercise training has been shown to stimulate muscle hypertrophy in middle and older aged men [37–41], although these studies also used some dietary manipulations. Taken together, this suggests the combination of T treatment and exercise training may confer additive benefits for lean body mass and some studies described below have directly addressed this question.

### The Impact of Testosterone on Lean Body Mass

Cross-sectional studies have associated higher endogenous T levels with higher lean body mass [42–47] and the majority of interventional studies in older men have reported that T improves lean body mass (Additional file 1: Table S1, mean effect across studies ~ 2.2 kg) [35, 48–60]. A study by Bhasin et al. [56] demonstrated that older men (60–75 years, *n* = 60) appeared to be as responsive as younger men (19–35 years, *n* = 61) to the anabolic effects of T treatment, assessed using dual energy x-ray absorptiometry (DXA) [56]. In older adults (≥ 65 years), an ‘umbrella review’ concluded that T administration was justified as a pharmacological intervention in men with low baseline T levels (6.9–10.4 nmol/L) to improve muscle mass [61]. It is pertinent to note that this review was not powered to assess any potential cardiovascular (CV) or cancer risks of T treatment in men and the use of T is still only indicated clinically for men with pathological androgen deficiency (disorders of the hypothalamus, pituitary or testes) [18, 62]. However, given the extensive evidence of the anabolic effects of T, coupled with the known association of low muscle mass and all-cause mortality in men [63], these findings may have important implications for men with reduced muscle mass resulting from their inability to exercise due to disease or disability.

### The Impact of Exercise Training on Lean Body Mass

There is a plethora of evidence demonstrating that exercise training can increase lean muscle mass across the lifespan, and in various disease states [64–70]. Given its association with all-cause mortality [31], maintaining or improving muscle mass becomes increasingly important into middle and older age [71]. Specifically in middle/older aged men (50–76 years), resistance training studies of 12–16 weeks report average lean body mass improvements of ~ 1.3 kg [37–41]. This result is in line with a meta-analysis by Peterson et al. [64] comprising 49 studies representing 1328 participants who were > 50 years. The authors concluded from their analysis that 20.5 weeks of resistance exercise training elicits an approximate 1.1 kg increase in lean body mass among older adults. It is important to note that the meta-analysis also included women, although sex was not significantly associated with the changes reported in lean body mass.

### The Impact of Combined Testosterone and Exercise Training on Lean Body Mass

A recent meta-analysis by Falqueto et al. [72] reported that the combination of T treatment and exercise training (duration 3–52 weeks) resulted in greater improvements in lean body mass compared to exercise training alone. However, of the 27 included studies (*n* = 1114) only one (*n* = 24) [73] was performed in healthy adult men, with the remainder involving clinical populations such as those with heart failure [74, 75], kidney failure [76], chronic obstructive pulmonary disease [77, 78], and spinal cord injury [79]. This highlights the paucity of data surrounding the effects of T and exercise training on aging in apparently healthy men, without major medical comorbidities.

Intervention studies in younger men report an additive effect on lean body mass when T treatment is combined with exercise training (Additional file 1: Table S2) [80–82]. Bhasin et al*.* [80] reported that, when 10 weeks of strength training was combined with higher doses (600 mg given intramuscularly on a weekly basis) of T in 43 men aged 19–40 years, lean body mass increased significantly (+ 6.1 kg) compared to men in the no-exercise groups (T alone: + 3.2 kg; placebo alone: + 0.8 kg). Exercise alone resulted in average lean body mass gains of 1.9 kg [80]. In men of similar age (*n* = 21, 19–45 years), Giorgi et al. [82] combined a supraphysiological T dose (3.5 mg/kg bodyweight weekly) with strength training over 12 weeks and reported significantly greater rectus femoris girth in the T + Ex group, compared with placebo. Similarly, a recent study by Pasiakos et al. [81] found that lean body mass improvements in young males (25 ± 5 years, *n* = 50) were significantly greater in those who received weekly T injections, compared to placebo (T + Ex: + 2.5 kg; P + Ex: -0.3 kg), after both groups completed 28 days of military-relevant exercise with a diet-induced energy deficit. The combination of T + Ex therefore appears to be additive for *younger* men with the magnitude of benefit ranging from ~ 2–5 kg over 10–12 weeks when supraphysiological doses are employed.

Studies assessing whether the addition of T treatment to an exercise training program in older men provides greater benefits for lean body mass than placebo have reported inconsistent results (Additional file 1: Table S2) [83–87]. Sullivan et al. [84] and Kvorning et al. [83] reported no additive effect of T + Ex on lean body mass following a 12-week resistance training intervention in older hypogonadal men. Further, Katznelson et al. [86] reported an absence of body composition change in either T treatment or exercise groups, or their combination, following a 12-week home-based Theraband exercise program. Conversely, in a group of healthy older men with low-normal T levels, Hildreth et al. [85] reported that the addition of T treatment to progressive resistance exercise training for 12 months led to greater improvements in lean body mass than exercise alone. Similarly, Barnouin et al. [87] reported the addition of T treatment to a lifestyle intervention provided superior lean body mass results to placebo in obese men (*n* = 83, > 65 years). Of note, in the Barnouin et al. [87] study, the addition of T treatment only attenuated the loss of lean body mass compared to placebo; lean body mass itself was not increased. Only one of the aforementioned studies employed a combined resistance and aerobic training program, which can be as effective as resistance training alone for improving lean body mass [88]. However, the lifestyle intervention in that study was also inclusive of caloric restriction (500–750 kcal/day deficit) which undoubtedly affected lean body mass.

In our recent 2 × 2 factorial trial comparing the impacts of T and exercise (TEX) [27–29], we randomised 80 men aged 50–70 years with waist girth ≥ 95 cm and low-normal serum T levels to transdermal T, or matching placebo (P) and to supervised center-based exercise training (Ex) or no additional exercise (NEx) [T + Ex group mean(95%CI) T levels nmol/L: 12.9(10.7–15.0); T + Nex 11.0(8.6–13.3); P + Ex 12.3(9.9–14.7); P + Nex 13.0(10.9–15.2)]. Post-intervention changes in T concentration were significant in the T treatment groups [T + Ex group 15.9(13.8–18.1); T + Nex 14.0(11.7–16.4)], but not in the placebo groups [P + Ex 13.2(10.6–15.7); P + Nex 12.1(9.9–14.3)]. The exercise intervention involved circuits of eight machine-based resistance exercises (leg press, chest press, seated calf raise, lat pulldown, leg curl, dual biceps curl, abdominal crunch, and triceps extension), alternated with eight aerobic cycling stations. Exercises were performed for 45 s, with 15-s intervals to facilitate movement between stations in the circuit. Resistance exercise intensity (2 sets of 12–15 reps, progressed to 3 sets at week 3) was initially performed at 65%1RM, progressed to 80% 1RM by weeks 4–6. Aerobic exercise was initially performed at 65% HRmax, progressed to 85%HRmax by weeks 4–6 on the cycle ergometer. Transdermal doses of T have to allow for limited absorption from normal skin, and in the absence of a depot tend to be given daily (46). The dose of T (100 mg applied to the upper body daily) was designed to raise T levels from low-normal to mid- or high-normal range in these participants, rather than to raise levels above the physiological range. We observed main effects of T treatment with increased total, leg and arm lean body mass (assessed by DXA) [28], thereby reinforcing the anabolic effects of T (see Fig. 2). Furthermore, we observed that T alone significantly increased leg lean body mass (+ 0.5 kg/2.3%) compared to placebo alone (− 0.4 kg/− 1.9%) over 12 weeks [28]. Given that muscle mass in the lower extremities is an important determinant of mobility status with aging [89, 90], our results may have implications for older men unable to exercise due to disease or disability. The 0.7 kg (1.1%) increase in total lean body mass in the exercise alone group was marginally lower than that reported (1.1 kg) in a meta-analysis of resistance exercise training studies in 1328 participants > 50 years [64], perhaps reflecting the shorter duration of our TEX study (12 weeks). Furthermore, our exercise intervention consisted of both aerobic and resistance training, making direct comparisons with the above meta-analysis difficult. Our findings are in agreement with previous T [35, 48–60] and exercise [37–41] studies in men as we report significant main effects of T (alone) and exercise (alone) to improve total lean body mass. Although we did not observe additive effects of T treatment and exercise to improve total lean body mass, we did observe that the combination had additive effects in specific regions (e.g. leg and arm lean body mass). Furthermore, the increase in total lean body mass was largest in men who received both T and exercise training [28]. Further studies are needed to examine this question, and to determine the effects of longer durations of these interventions.

### Summary: Effects of Testosterone, Exercise, and Their Combination on Lean Body Mass

**Fig. 1:**
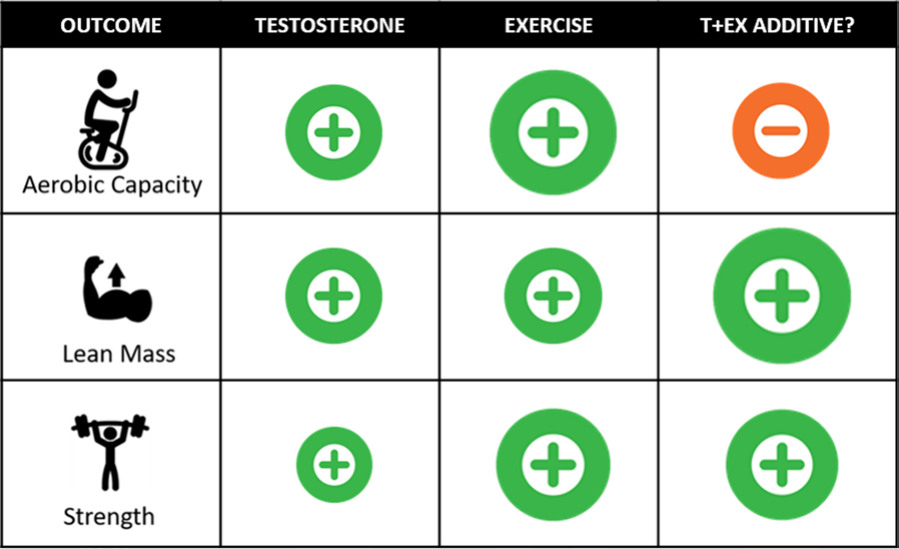
Summary of trials which have independently modified T levels within the physiological range, or utilised exercise training interventions, or combined these interventions. Where a positive impact is indicated for T or exercise, there is typically evidence that the benefits are dependent on dose, and in the case of T, also on route of administration (favouring intramuscular injection). Some evidence suggests that additive effects for lean body mass may be more apparent in younger men, but that gains in strength may be less age-dependent, possibly due to neural benefits. Interpretation of this disparate literature should be compared to the results of an RCT comparing exercise and T effects, summarised in Fig. 2

**Fig. 2:**
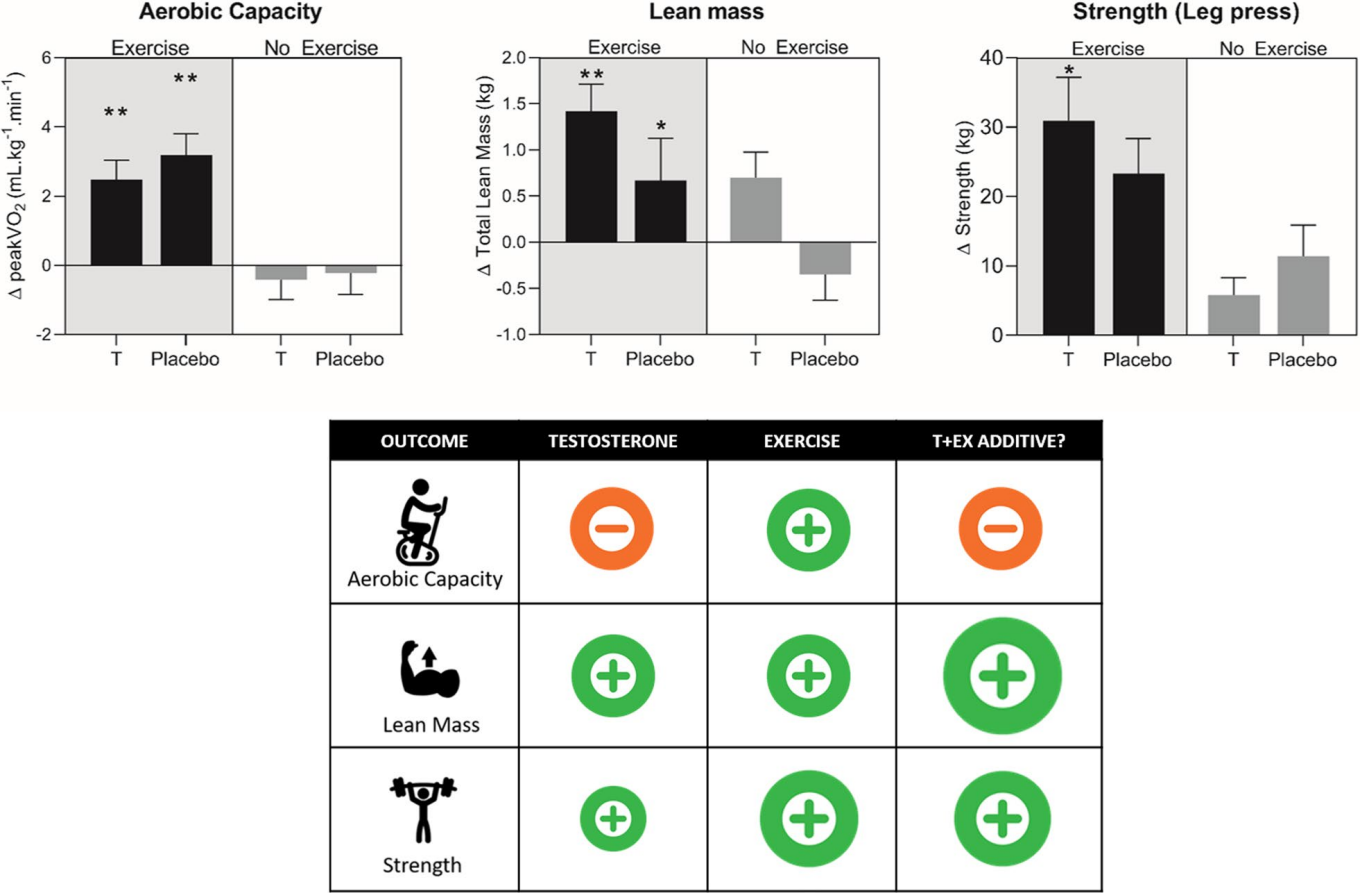
Summary of results from the 2 × 2 factorial trial of Testosterone and Exercise (TEX) which directly compared the combined and independent impacts of T treatment (in the physiological range) and supervised center-based exercise. Upper: Changes from baseline following the 12 week intervention, **P < 0.001, *P < 0.05 for week 12 change from baseline compared with placebo + no exercise group change. Lower: Infographic summarising the results from our TEX trial. T + Ex column indicates whether the addition of testosterone (T) to exercise training (Ex) provides additive benefit compared to exercise alone. ‘ + ’ indicates improvement, ‘–’ indicates no significant change

There is clear evidence that both T treatment and exercise improve lean body mass in healthy older men (Fig. 1). If improvement in lean body mass is the primary aim, then T supplementation could be considered, and the combination of T and exercise may be more beneficial than either in isolation (Figs. 1 and 2). However, the effects of T (and exercise) should be reviewed in the overall context of cardiovascular risk (see section below: *Are There Cardiovascular Risks Associated With Testosterone Treatment?*). Men who are physically able to exercise safely should be encouraged to do so, not only in terms of building lean body mass but for the myriad of other health benefits exercise training offers [91–93]. Finally, it has been reported that increases in skeletal muscle mass as a result of T treatment may be dose-dependent [50, 52, 56, 94, 95]. It is therefore of interest in our TEX study, that despite a much lower (physiological) T dose delivered in older participants, we observed improvements in lean body mass.

### Muscular Strength: The Effects of Testosterone, Exercise, and Their Combination

Although inter-related, a change in lean body mass is not always an indicator of functional (strength) change [96, 97] (and vice versa), and there are inconsistent reports regarding the association of the increase in lean body mass following T treatment and changes in muscular strength and performance [49, 51, 53, 55, 98]. It has been reported that a decline in muscular strength is more indicative of functional decline than decrease in muscle mass [99]. Loss of muscular strength with increasing age is associated with decreases in function, the ability to conduct activities of daily living, and independence [100, 101]. Consequently, low muscle strength also independently predicts all-cause mortality in men [102–105], making it a pertinent outcome to assess in middle to older aged men.

### The Impact of Testosterone on Muscular Strength

Data from cross-sectional comparisons suggest that lower endogenous T levels may be associated with lower levels of muscular strength in younger [106], middle aged [107] and older [108] men. However, data from interventional studies in older men are less conclusive, possibly due to differences in study duration, or T dose and/or route of administration (see Additional file 1: Table S3). Some T intervention studies in older men have reported no difference in muscular strength between T-treated and placebo groups [49, 51, 53, 55, 58, 109–113]. In contrast, other studies of T treatment alone (i.e. no exercise intervention) have reported improvements in strength [35, 48, 52, 57, 59, 98, 114, 115]. However, of the studies that have reported improvements in muscular strength, one did not include a control group [52], three reported only hand-grip measurements as a surrogate of strength [57, 98, 115], three were limited by small sample sizes (*n* < 12 per group) [35, 59, 114], and another was conducted in older frail men [48]. Furthermore, a meta-analysis of 1083 males aged 50–78 years across 29 RCTs reported only a small effect size of 0.3 for dominant knee extension and hand-grip strength [116]. In contrast, a more recent meta-analysis of 41 RCTs in middle-aged and older men reported a large effect size of 0.9 for total body strength following T treatment [117]. The authors also concluded that studies employing intramuscular T injection resulted in an 11.2% increase of total body strength, in comparison to transdermal preparations, which improved strength by 2.1%.

### Is an Increase in Strength Following Testosterone Treatment Dose-Dependent?

Given that T intervention studies employ a range of doses, Bhasin et al. [56] used a long-acting gonadotropin-releasing hormone agonist to suppress endogenous T secretion and assessed responses to five different doses of T (ranging from 25 to 600 mg/wk) over five months to determine T dose–response relationships in younger (19–35 years, *n* = 61) and older (60–75 years, *n* = 60) men. Dose-dependent increases in leg press strength were elicited in both younger and older men, with no effect of age [56]. These results highlighted that older men were as responsive as younger men (age effect: *P* = 0.84) to the beneficial effects of T on strength (assessed via leg press). In addition, the authors concluded the best ‘trade-off’ was achieved with a T dose of 125 mg per week given intramuscularly when improvements in lean body mass, muscle strength and frequency of adverse events were taken into consideration. Although perhaps unsurprising, it is important to note that, following cessation of T treatment, muscular strength levels may not be maintained (unless otherwise targeted) [118], and the rate of strength loss may be correlated with the length of the antecedent T administration period [82].

### The Impact of Exercise on Strength

Low muscle strength is independently associated with an increased risk of all-cause mortality, independent of muscle mass [119]. However, the effect of resistance training on muscular strength in older adults is well established [120, 121] and supported by the highest category of evidence [122, 123]. A systematic review of 41 progressive resistance strength training trials in older adults (*n* = 1955) reported a moderate-to-large beneficial effect on strength (0.68) [124]. These results were later reinforced by a Cochrane review of 73 trials comprising 3059 older adults which demonstrated that progressive resistance training has a large effect (0.84) on muscle strength [121]. Further, a meta-analysis of 47 studies representing 1079 participants reported that older adults can achieve substantial muscular strength gains in major muscle groups (24–29% improvement across leg press, chest press, knee extension and latissimus-pulldown exercises) following resistance training [120].

### Can Exercise Training Modify Age-Related Decline in Strength?

A reduction in size and number of muscle fibres, specifically in type II (fast-twitch) fibres, underpins age-associated strength decreases [125]. The selective atrophy of type II muscle fibres with age decreases the maximum relaxation rate [126], which can be ascribed to decreased sarcoplasmic reticulum activity and reduced sliding speed of actin on myosin [127, 128]. A gradual decrease in the number of muscle fibres begins during the 5th decade of life with an approximate 50% reduction by 80 years [129]. However, a slowing of this process is evident in those who remain physically active into older age—e.g. masters athletes [130, 131]. There is also evidence to show that six months of strength training in elderly men can induce lower limb strength increases similar in relative terms to men 30 years their junior (40 year old increase: 22% *vs* 70 year old increase: 21%).

Although a portion of the age-related decline in strength may be related to muscle mass, there is also evidence to suggest that neural processes play a role. Specifically, motor unit losses of 47% have been reported in older (60–81 years) compared to younger (22–38 years) participants [132]. Although some will atrophy, denervated muscle fibres may also be adopted by other surviving motor neurons, resulting in larger motor units [133]. Whether chronic exercise training can delay the decrease in motor unit numbers associated with aging remains unclear [134, 135].

### The Impact of Combined Testosterone Treatment and Exercise Training on Strength

Intervention studies combining T treatment and exercise have produced heterogenous results in terms of muscular strength outcomes, which may be attributed to distinct T doses, routes of administration and/or exercise training programs (see Additional file 1: Table S4). The combination of supraphysiological T doses and resistance exercise training has been shown to increase muscular strength more than exercise alone following 10–12-week interventions in healthy young men (aged 19–45 years) [80, 82]. Specifically, using 600 mg/wk intramuscular injections, Bhasin et al. [80] reported increases in 1RM of 38% in the T + Ex group compared to those that performed exercise alone (11%). In this study, serum T levels in the T + Ex group increased from 14.9 ± 1.3 at baseline to 112.5 ± 10.6 nmol/L. Similarly, Giorgi et al. [82] administered 3.5 mg/kg/wk injections (average bodyweight: 83 kg = 290 mg/wk) and reported increases of 22% and 9% in the T + Ex and P + Ex groups respectively (changes in T levels were not reported). Collectively these studies suggest the addition of supraphysiological T treatment to exercise training over 12 weeks improves the benefit of exercise alone by ~ 2–threefold. In contrast, another study of men aged 18–39 years by Pasiakos et al. [81] failed to show any difference in lower body muscular strength changes between T (200 mg/wk) and placebo groups after 28 days of military-relevant exercise. They observed changes in T from 15.5 to 36.2 nmol/L in the T group during this time. However, direct comparisons between Pasiakos et al. [81] and the earlier studies from Bhasin et al*.* [80] and Giorgi et al. [82] are difficult, as all participants from Pasiakos et al. [81] also underwent a severe (55%) energy deficit (diet and exercise induced) which likely affected muscular strength improvements. It is also important to highlight that the exercise training regime implemented by Pasiakos et al. was relatively short (28 days) and primarily aerobic-based to “*reflect the aerobic-type physical work that occurs during sustained, strenuous military operations*”, which may account for the lack of lower body muscular strength improvement reported.

Studies combining T treatment and exercise training in older men with low-normal baseline T levels have failed to show additive muscular strength benefits over 3–12 months (Additional file 1: Table S4) [83–85, 87]. Sullivan et al. [84] reported a trend toward greater strength improvement in 1RM bench press with T (100 mg injections) over 12 weeks in 71 men aged 65–93 years but these results did not achieve statistical significance. Of note, although significantly greater increases in mid-thigh cross-sectional area were reported for T + Ex, this did not translate into greater strength improvements in 1RM leg press, reinforcing the distinction between lean body mass and strength improvements. Kvorning et al. [83] also failed to show any additional benefit of adding 50 mg of transdermal T (gel) to 12 weeks of strength training in a smaller study (T + Ex: *n* = 6, P + Ex: *n* = 8) of men aged 60–78 years. In a 12-month study of 167 men > 60 years by Hildreth et al. [85], the addition of T to supervised progressive resistance training did not significantly affect any 1RM strength measures. Of note however, the improvement in upper body strength in the non-exercisers was significantly greater in those randomised to T, compared to placebo. In line with these results, Barnouin et al. [87] also failed to show any significant 1RM strength differences between T and placebo in 83 obese men (> 65 years, body mass index [BMI] > 30 kg/m^2^) when both groups also engaged in a lifestyle intervention inclusive of caloric restriction and exercise training.

All studies in middle-aged and older men have employed training programs that were predominantly resistance-based. However, interventions that are specifically designed to target both aerobic and muscular components of health may be more beneficial than either modality in isolation, particularly for improvement in tasks of daily living [88, 136]. Contrary to widely held belief, endurance trained older men (70–81 years) also have preserved strength characteristics relative to body mass [131]. In the TEX study which involved combined resistance and endurance training, we did not observe any main effects of T on strength measures [28] (Fig. 2). As muscular strength responses to T may be dose-dependent [56] and further influenced by route of administration [117], we cannot exclude the possibility that a higher dose, alternate route of T administration, or longer treatment may have yielded different results. Conversely, we reported main effects of exercise training which increased all strength measures [28]. These findings suggest that the addition of T (targeting physiological levels) to an exercise program might not provide further benefit in terms of muscular strength than exercise alone. This indicates that middle and older aged men with low T levels are likely to benefit from an exercise program targeted at improving muscular strength, which in turn would be expected to slow age-related declines in function and preserve ability to conduct activities of daily living independently [100, 101]. Although our results indicated a potential additive effect of T and exercise on lean body mass (Fig. 2), this did not translate into strength gains, which is in line with some previous literature.

### Summary: Effects of Exercise Training and Testosterone on Strength in Middle and Older Aged Men

The likelihood of detecting significant differences in strength between T treatment and placebo tends to rise only if the dose is > 125 mg/wk (usually delivered via intramuscular injection). Studies that have used lower doses or different formulations have typically failed to report significant T effects. The literature concerning the effects of exercise training on muscular strength in older men is more homogeneous; exercise training improves muscular strength in healthy middle aged and older men. There is evidence to suggest that the addition of supraphysiological T treatment to an exercise training program is ~ 2–3 × more beneficial for muscular strength than placebo in men. However, it should be emphasized that important clinical concerns have been raised regarding the use of supraphysiological T doses in older men [56] (see section below: *Are There Cardiovascular Risks Associated With Testosterone Treatment?*), and no study (including TEX) has replicated this finding using doses of T that target the physiological range. For men motivated to maintain/build muscle strength into older age, an exercise program is likely to be more beneficial than exogenous T treatment. The addition of physiological T treatment does not appear to provide any benefit beyond that of exercise alone.

### Aerobic Capacity: Effects of Testosterone, Exercise, and Their Combination

Exercise capacity provides an index of integrative human functional capacity which predicts all-cause and cardiovascular mortality [137–139]. Every 1 metabolic equivalent improvement in aerobic fitness is associated with 15% and 19% lower risk of all-cause and CVD mortality respectively [140]. With aging, aerobic fitness tends to decline, with decreases of 15–20% reported in men during the fifth and sixth decades of life [15].

### The Impact of Testosterone Treatment on Aerobic Fitness

Short-term T supplementation has been shown to improve total exercise time in men with coronary artery disease [141, 142], and also to improve VO_2_peak in men with heart failure [143]. However, longer-term T studies (6–24 months) in men without CVD have reported heterogeneous results concerning measures of peak aerobic exercise performance (see Additional file 1: Table S5) [111, 112, 144–146]. In a group of 64 mobility-limited, older men (65–86 years), Storer et al*.* [144] reported a significant difference in aerobic capacity between T treatment and placebo groups following six months of 100 mg daily transdermal T supplementation. Although the relative increase in VO_2_peak with T treatment was modest (0.83 mL/kg^/^min), the significant between-group difference was attributed to the greater than expected decline in the placebo group (− 0.89 mL/kg^/^min). These findings were reinforced by a later study by Traustadottir et al*.* [145] who reported that 3 years of 75 mg daily transdermal T treatment attenuated the expected age-related decline that was observed in the placebo group (average 3-year decrease, 0.88 mL/kg^/^min). In line with these findings, Blackman et al. [112] reported a significant difference between T (biweekly 100 mg injection) and placebo groups in men aged 65–88 years (*n* = 38) following a six-month intervention. In contrast to these studies, Nair et al*.* [146] concluded that there was no significant difference in aerobic capacity between the T and placebo groups after 2 years of T treatment (5 mg transdermal patch per day) in ~ 58 older men. However, in this study, the median VO_2_ peak data at baseline were 40.7 and 40.4 mL/kg^/^min (in the T and placebo groups respectively) which places these men at the 90th percentile for males aged between 60 and 69 years [147]. It is conceivable that these high VO_2_peak levels at baseline may have precluded the study’s capacity to demonstrate improvement [148]. It is also important to note that the T dose administered in the Nair et al*.* [146] trial was modest (5 mg) and that the same (5 mg transdermal patch) dose was used by Giannoulis et al. [111] who also failed to report any significant difference between T and placebo groups following a 6-month intervention in 43 men aged 65–80 years. Taken together, these studies in healthy middle-aged and older men suggest moderate effects of T to improve aerobic capacity, with subtle between-group differences contributed to by the decline observed in placebo groups (Additional file 1: Table S5).

### Mechanisms Linking Testosterone and Aerobic Fitness

The mechanistic pathways by which T affects VO_2_peak are not yet fully understood and are likely multifactorial. T treatment in men has been associated with improved oxygen delivery and utilization through increases in hemoglobin [149], muscle capillarisation [150], and size of type I muscle fibres [151]. Of note, both younger and older men have been shown to increase red cell mass in a dose-dependent manner following 20 weeks of T treatment [149]. Given O_2_ transport capacity correlates directly with aerobic performance [152], it is surprising that there is no conclusive evidence regarding the effect of T treatment for improving VO_2_peak in middle-to-older aged men with low-normal serum T levels, and hence new RCTs are required.

### The Impact of Exercise Training on Aerobic Fitness

In apparently healthy middle aged and older men, studies assessing the impact of exercise training on VO_2_peak report improvements of 10–32% following 2–12 months of exercise training [153–158]. Improvements in VO_2_peak following exercise training may arise from central and/or peripheral adaptation. Centrally, modifications in cardiac structure and function [159–161] and heart rate have been reported following exercise training. Peripherally, improvements in skeletal muscle structure and function [162, 163], mitochondrial density [164, 165], and decreased peripheral vascular resistance have also been identified as mediators that underpin improvements in oxygen delivery and utilization following exercise training [166–171]. A recent systematic review by Montero et al. [172] comprising 16 endurance training studies (total *n* = 153 primarily untrained healthy participants, 81% male, mean age 42–71 years) assessed the relative impact of changes in maximal cardiac output and arteriovenous oxygen (*a*-*v*O_2_) difference on maximal oxygen consumption (VO_2_max). The authors concluded that although both cardiac output and *a*-*v*O_2_ difference improved with exercise training, the improvement in VO_2_max was more attributable to the change in cardiac output, based on the linearity and strength of the relationship between the latter variables. Given that maximal HR is unchanged following exercise training, the increase in cardiac output following training derives from an increase in maximal stroke volume. Mechanisms responsible for the increased stroke volume may relate to enhanced left ventricular structure/function [160] and/or expanded blood volumes [173, 174].

### Does the Combination of Exercise Training and Testosterone Have Additive Impacts on Aerobic Fitness?

Few interventional studies have assessed the effect of combining T treatment and exercise on functional capacity in healthy older men, or effects on a measure of functional capacity. In 167 healthy older (66 ± 5 years) men with low-normal T levels (7–12 nmol/L), Hildreth et al. [85] reported that neither T treatment, exercise, nor their combination, improved results in the six-minute walk test. However, the exercise intervention employed by Hildreth et al*.* [85] was predominantly resistance-based, which may have reduced the likelihood of observing improvements in aerobic measures [175]. In contrast, Barnouin et al. [87] reported that the addition of T treatment to a lifestyle intervention (caloric restriction and exercise training) significantly improved VO_2_peak compared to placebo (T: + 4.0 vs. P: + 2.9 mL/kg^/^min; T: + 0.42 vs. P: + 0.29 L^/^min) in older, obese, hypogonadal men with mild-moderate frailty (*n* = 83, > 65 years, BMI > 30 kg/m^2^, T < 10.4 nmol/L). However, the study by Barnouin et al. [87] did not include a control group, which prevents definitive conclusions regarding the impacts of T and exercise on aerobic fitness.

Consistent with previous literature, we recently observed that T treatment at a physiological dose did not influence changes in VO_2_peak in the T alone group [28] (i.e. no exercise intervention) (Fig. 2) [111, 112, 144–146]. In contrast, but also in line with previous research [153–158], we reported a 13% (3.2 mL/kg^/^min) increase in VO_2_peak in the exercise only group [28] and a 10% gain in the T + Ex group (2.5 mL/kg^/^min) (Fig. 2). These results highlight that exercise training is superior to T treatment for improving VO_2_peak in middle and older aged men with low-normal T levels. The significant main effect of exercise was also maintained when calculated in absolute terms (L/min).

### Summary: Effects of Exercise and Testosterone on Aerobic Fitness

The literature to date suggests that differences between T and placebo groups are largely attributed to the prevention of time-related decline in placebo groups. In contrast, there is a mature evidence-base which supports the role of exercise in improving aerobic fitness. Our results [28], and others, suggest that the addition of T at a physiological dose neither increases nor diminishes the effect of exercise on VO_2_peak. Therefore, in order to improve aerobic capacity in middle-aged and older men, exercise training should be recommended and implemented. The addition of T treatment at a physiological dose does not appear to provide any benefit beyond that of exercise alone, at least over a short timeframe of intervention.

### Are There Cardiovascular Risks Associated With Testosterone Treatment?

The literature reviewed in the preceding sections suggests that higher doses of T, particularly if delivered via intramuscular injection, may have larger effects on skeletal muscle. However, this must be balanced against the potential harms of T delivered at higher does than those required to achieve physiological concentrations. An RCT of transdermal T at relatively higher doses in 209 men aged 65 years and older with mobility limitations was discontinued due to an excess of broadly defined cardiovascular adverse events in the active treatment arm of the study [176]. However, a similar trial of transdermal T using conventional doses in 274 men aged 65 and older who were frail or intermediate-frail, found no increase in cardiovascular adverse events in T-treated men [48]. Contemporary meta-analyses of RCTs have not associated T treatment with increased risk of cardiovascular adverse events [177, 178]. In the Testosterone Trials (T Trials), a large RCT of 788 men aged 65 years and older randomised to T treatment or placebo for 12 months, 7 men in the T arm, and 7 in the placebo arm, experienced a major adverse cardiovascular event [179] (MACE, comprising myocardial infarction, stroke or death from cardiovascular causes). The cardiovascular sub-study of T Trials analysed 73 men from the T arm and 65 from the placebo arm of the main trial, reporting an increase in non-calcified plaque volume in T-treated men, with larger and longer duration studies recommended to clarify this issue [180, 181]. The Testosterone For Prevention of Type 2 Diabetes Mellitus trial randomised 1,007 men aged 50–74 years, with waist circumference 95 cm or greater and either impaired glucose tolerance or newly diagnosed diabetes, to intramuscular T decanoate versus placebo for 2 years, on a background of lifestyle intervention [182]. In T4DM, T treatment reduced the risk of type 2 diabetes at 2 years by 40%, with 17 men in the placebo arm and 12 in the T arm experiencing a MACE during the trial [183]. Therefore, while the possibility that T treatment, particularly at higher doses in older men, might be associated with cardiovascular adverse events has been raised, results from meta-analyses and from two recent large RCTs provide some reassurance. Nevertheless, none of the preceding RCTs were powered for MACE as a pre-specified outcome. The results of TRAVERSE, an FDA-mandated cardiovascular safety trial of transdermal T, are a major advance in this area [184]. TRAVERSE enrolled 5246 men aged 45–80 years with cardiovascular risk factors or disease, and low-normal T concentrations. Men were randomised to T vs placebo, remaining on treatment on average for 22 months, with follow-up for an average of 33 months. Of the T-treated men, 182/2596 (7%) experienced the primary safety endpoint (first occurrence of death from cardiovascular causes, nonfatal myocardial infarction, or nonfatal stroke) vs 190/2602 (7.3%) of men in the placebo group (HR 0.96, 95% CI 0.78–1.17) [185]. Likewise, rates of the secondary endpoint (first occurrence of death from cardiovascular causes, nonfatal myocardial infarction, nonfatal stroke, or coronary revascularization) were similar in T and placebo groups, i.e. 269 (10.4%) vs 264 (10.1%) (HR 1.02, 95% CI 0.86–1.21), as was all-cause mortality risk (5.5% vs 5.7%; HR 0.98, 95% CI 0.78–1.23). TRAVERSE provides reassurance as to the cardiovascular safety of T treatment in middle-aged to older men with cardiovascular risk factors or disease.

## Conclusions

Older men often exhibit lower T concentrations compared with younger or middle-aged men [1, 2, 5, 9]. However, it remains unclear whether low T is an inevitable consequence of aging, or a reflection of comorbidity accumulation (e.g. obesity, chronic diseases) throughout the aging process. Healthy men aged 40–69 years can have stable T concentrations over a 4-year period of follow-up [186]. However, above the age of 70 years, longitudinal declines in T levels are accompanied by increases in luteinising hormone, suggesting impairment of testicular hormone production [10]. Furthermore, the majority of people aged ≥ 65 years do not meet physical activity guidelines [187].

T is an effective physiological countermeasure for loss of lean body mass and strength in men with androgen deficiency due to disorders of the hypothalamus, pituitary or testes, who cannot produce sufficient endogenous T. Whether it represents a viable intervention to increase lean body mass and strength, and/or aerobic fitness in middle-aged to older men *without* disorders of the hypothalamus, pituitary or testes, remains to be established. Nonetheless, this review highlights that exercise should be a first line strategy to improve strength and aerobic fitness in aging men and that combined (aerobic and resistance) exercise training programs in middle and older-aged men are beneficial. A caveat is that many of the studies we reviewed addressed the impacts of exercise when it was center-based, supervised and verified, rather than community or home-based. An ongoing challenge with translating such benefits is adherence with longer-term exercise in community and home-based settings.

In the longer term, improvements in body composition and in strength and aerobic fitness are likely to have important consequences for successful aging. Our review suggests that T has impacts on strength, body composition and aerobic fitness outcomes that are dependent upon dose, route of administration, and formulation. Whilst T treatment in middle-aged and older men can improve lean body mass, exercise training enhances lean body mass, aerobic fitness and strength. Future research should address whether, for those unable to exercise, benefit accrues from T treatment to maintain muscle mass and avoid frailty-related health sequelae.

### Supplementary Information

**Additional file 1:** Contains detailed tables comparing the impacts of testosterone and exercise training on lean mass, strength and fitness in aging men.

## Data Availability

Not applicable in this narrative review.

## Abbreviations

T: Testosterone
Ex: Exercise
T + Ex: Testosterone plus exercise
T + NEx: Testosterone plus no exercise
P + Ex: Placebo plus exercise
P + NEx: Placebo plus no exercise
PA: Physical activity
AAS: Anabolic androgenic steroid
TEX: Testosterone and exercise study
DXA: Dual energy X-ray absorptiometry
CV: Cardiovascular
CVD: Cardiovascular disease
1RM: 1 Repetition maximum; a measure of maximal strength
HRmax: Maximal heart rate
RCT: Randomised controlled trial
VO_2_peak: Peak oxygen uptake
MACE: Major adverse cardiovascular events
FDA: Federal Drug Administration
