# Supplementary material for: Comparing the Impacts of Testosterone and Exercise on Lean Body Mass, Strength and Aerobic Fitness in Aging Men
Source: Sports Med Open. 2024 Apr 2;10:30. doi: 10.1186/s40798-024-00703-x (PMC10987448; doi:10.1186/s40798-024-00703-x)
Supplement: Supplementary file 1 — Additional file 1: Contains detailed tables comparing the impacts of testosterone and exercise training on lean mass, strength and fitness in aging men. [file 40798_2024_703_MOESM1_ESM.docx]

***Supplementary Material - Exercise and Sports Sciences Reviews (ESSR): Brief Review***

**Testosterone versus exercise: Which intervention is best for lean mass, strength and fitness in aging men?**

Daniel J. Green^1*^, Lauren C. Chasland^1,2*^, Bu B. Yeap^3,4^, and Louise H. Naylor^1,2^

*Contributed equally to authorship

^1^School of Human Sciences (Exercise and Sport Science),

The University of Western Australia, Perth, WA, Australia;

^2^Allied Health Department, Fiona Stanley Hospital, Perth, Western Australia;

^3^Medical School, University of Western Australia, Perth, Western Australia;

^4^Department of Endocrinology and Diabetes, Fiona Stanley Hospital,

Perth, Western Australia

**Supplementary Material**

**Table 1:** Studies investigating the effect of testosterone treatment on lean mass in healthy middle-aged and older men.

**Table 2:** Studies investigating the effect of testosterone treatment and exercise on lean mass in healthy young and older men.

**Table 3:** Studies investigating the effect of testosterone treatment on muscular strength and function in healthy middle-aged and older men.

**Table 4:** Studies investigating the effect of testosterone treatment and exercise on strength in healthy young and older men.

**Table 5:** Studies investigating the effect of testosterone treatment on VO_2_peak in healthy middle-aged and older men

**Table 1:** Studies investigating the effect of testosterone treatment on lean mass in healthy middle-aged and older men.

| **Source** | **Study  design** | **Intervention** | **Primary outcome** | **Body composition method** | **Lean mass results** | **Limitations** |
| --- | --- | --- | --- | --- | --- | --- |
| Borst *et al*. (1) (2014) | TYPE: Randomized, placebo-controlled  DURATION:  52 weeks  PARTICIPANTS:  30 men aged >60 yr, with a serum total T <10.4 nmol·L^-1^ | Testosterone: 125 mg·wk^-1^ intramuscular (im) injection  Electrochemoluminescence immunoassay.  Baseline 8.5 ± 2.5 nmol·L^-1^;  0-12mo change 7.9 (-0.9 to 16.8) nmol·L^-1^ | Muscle strength | DXA | Main effect of T (*P =* 0.032).  - T increased fat free mass by 5.7 kg. | - Men only excluded if received T within 4 weeks. - 33% attrition, low completion numbers (T: *n* = 7, P: *n* = 12) |
| Behre et al. (2) (2012) | TYPE:  Randomized, double blinded,  placebo-controlled  DURATION:  26 weeks  PARTICIPANTS:  362 men aged 48 – 80 yr with low-normal total serum testosterone (<15.0 nmol·L^-1^) | Testosterone gel  (5 g, equivalent to 50 mg testosterone) or placebo gel (5 g)  T (ECLIA) T group:  Baseline 10.4 ± 2.6 nmol·L^-1^,  P group: 10.6 ± 2.6 nmol·L^-1^  6 month values: T group 20.45; P group 3.43 nmol·L^-1^ | Body composition | DXA | Significant difference between T and *P* <0.01). LBM increased 1.2 kg in T group and no change in P group. | - 31 T-treated men did not complete study due to increases in PSA - Conducted across 8 countries - Men excluded if BMI >35 kg·m^-2^ |
| Frederiksen *et al*. (3) (2012) | TYPE:  Randomized, double blinded,  placebo-controlled  DURATION:  26 weeks  PARTICIPANTS: 38 men aged 60-78 yr with waist circumference >94 cm and T level <7.3 nmol·L^-1^. | 5 g gel, containing 50 mg testosterone.  Liquid chromatography tandem mass spectrometry.  Baseline T group 12.5 ± 4.0  6 Months 22.2 ± 10.8 nmol·L^-1^;  P group 12.7 ± 4.4 to 10.2 ± 3.2 nmol·L^-1^ | Insulin sensitivity | DXA | Significant difference between T and P <0.01). LBM increased 1.7 kg in T group and decreased 0.1 kg in P group. | - 49 adverse events during study, 2 serious adverse events. One (venous thrombosis) was possibly treatment related; second was unrelated (car accident). |
| Srinivas-Shankar *et al.* (4) (2010) | TYPE:  Randomized, double-blind, placebo-controlled, parallel group, single-centre  DURATION:  26 weeks  PARTICIPANTS: 274 community-dwelling intermediate-frail and frail elderly men >65 yr with total T ≤12 nmol·L^-1^. | Transdermal T (50 mg·d^-1^) or  placebo gel  Chemiluminescent immunoassay. Baseline - T group 10.9 ± 3.1  P group 11.0 ±3.2 nmol·L^-1^.  6 mo – T group 18.4 ± 9.2, P group 10.7 ± 3.5 nmol·L^-1^ | Isometric knee extension peak torque  and isokinetic knee extension peak torque | DXA | LBM increased in the T group (*vs* placebo) with a mean  difference between groups of 1.1 (95% CI = 0.6 –1.5 kg; *P <* 0.001). | - Single serum T measurement to determine eligibility - Diet and physical activities of the participants were not standardized - Time gap between randomization and baseline assessment - Twelve randomized men withdrew before baseline assessment and before they received the allocated treatment. |
| Sattler *et al*. (5) (2009) | TYPE:  Randomized, controlled, double-blinded  DURATION:  16 weeks  PARTICIPANTS: 39 participants aged 65-90 yr with T (150–550 ng·dL^-1^; 5.21–19.1 nmol·L^-1^) | 5 g or 10 g of 1% testosterone transdermal gel was applied each morning for 16 wk  T was  measured by liquid chromatography tandem mass spectrometry assay. 5g group baseline T was 13.4 ±3.7 nmol·L^-1^, 10g group was 12.1 ± 0.3 nmol·L^-1^. | Body composition | DXA | 5 g·d^-1^ T increased lean body mass by 0.7 kg, 10 g·d^-1^ increased lean body mass by 1.5 kg. | - No true control group (all received either T or rhGH), - All subjects were treated monthly from baseline to week 12 using a Leydig cell clamp with a long-acting GnRH agonist (leuprolide acetate depot, 7.5 mg im) - T measured using immunoassay |
| Emmelot-Vonk *et al.*(6) (2008) | TYPE:  Double-blind, randomized, placebo controlled  DURATION:  26 weeks  PARTICIPANTS: 237 healthy men 60 - 80 yr with testosterone  <13.7 nmol·L^-1^ | 2 capsules  of 40 mg testosterone undecenoate  twice per day with breakfast and dinner  (equalling a total dose of 160 mg·d^-1^  of testosterone undecenoate), or matching  placebo  T (chemiluminescent enzyme immunoassay) in T group: 11.0 ± 1.9 nmol·L^-1^, P group 10.4 ±1.9. At 6mo, T was unchanged from baseline in the T group and increased slightly in the P group. Group difference at 6 mo was -3.2 nmol·L^-1^. | Functional mobility (Stanford Health Assessment Question-naire,  timed get up and go test, strength: isometric handgrip, isometric leg extension | DXA | Total body lean mass in the testosterone  group increased significantly  relative to the placebo group (T: +1.1 *vs* P: -0.3 kg, P < 0.001). | - Placebo group increased T levels more at 6 months than the T group - Oral capsules used. |
| Storer *et al.* (7) (2008) | TYPE:  Double-blind, randomized study  DURATION:  20 weeks  PARTICIPANTS Community-dwelling healthy men aged 60-75 yr (*n* = 44) | Monthly treatment with a gonadotropin-releasing hormone agonist plus 25, 50, 125, or 300 mg·wk^-1^ of intramuscular injections of testosterone enanthate.  Testosterone (immunoassay) pre-post values:  12.9 ± 4.4 to 6.1 ± 1.2; 11.4 ± 2.7 to 9.5 ± 0.6; 13.5 ± 4.5 to 29.6 ± 3.9 and 11.1 ± 3.8 to 61.9 ± 6.0 nmol·L^-1^ for the 25-, 50-, 125- and 300-mg·wk^-1^ dose groups respectively. | Skeletal muscle mass and strength | DXA | Skeletal muscle mass increased significantly from baseline in both the 125 (8.5 ± 4.0%) and 300 (11.1 ± 4.3%) mg·wk^-1^ dose groups. Skeletal muscle mass index (skeletal muscle mass divided by height squared) also increased dose-dependently with testosterone administration (*P* < 0.0001). | - No control / placebo group - 600 mg·wk^-1^ group was discontinued after higher number of adverse events in the 6 participants randomized to that group (data not included). |
| [Giannoulis](javascript:;) *et al*. (8) (2006) | TYPE:  randomized, double-blind, placebo-controlled  DURATION:  26 weeks  PARTICIPANTS:  43 healthy, community-dwelling, older men (age, 65–80 yr). | Testosterone (5 mg) or placebo was given by transdermal patches.  T (ADVIA Centaur analyzer) at baseline was 17 ± 1.2 nmol·L^-1^ for T group and 15 ±1.1 for P group; post intervention, T group 25 ± 3.3 nmol·L^-1^; no change P group. | Body composition | DXA | No significant differences between T and placebo in LBM | - 30% developed skin irritations from T patches - Immunoassay used for measurement of T (instead of LC/MS). - VO_2_peak and strength assessments conducted during same testing session. |
| Page *et al*. (9) (2005) | TYPE:  Randomized, double blinded,  placebo-controlled  DURATION:  156 weeks (3 yr)  PARTICIPANTS:  70 men aged 65-83 yr with T <350 ng·dL^-1^ (12.1 nmol·L^-1^) | 24 subjects received T only [T enanthate (TE);  200 mg im injection every 2 weeks, plus a daily oral placebo; 24 subjects received  placebo (sesame oil placebo), 1 mL im every 2 weeks, plus daily oral placebo.  T (via fluoroimmunoassay): T group 9.9 ± 1.6, P group 10.5 ± 1.7 nmol·L^-1^ at baseline. Total T significantly increased for T but not P group over the treatment period. | Physical function | DXA | Significant difference between T and P. T increased LBM by 3.7 kg, P decreased by 0.3 kg, *P* < 0.001) | - NB: An additional 22 subjects received finasteride (F; which partially blocks the conversion of T to dihydro-testosterone). LBM results of T+F were similar to that of T alone. - 29% attrition rate - Men asked not to engage in regular exercise for 36 months. |
| Wittert *et al.* (10) (2003) | TYPE: Randomized, double-blind, placebo-controlled  DURATION:  52 weeks  PARTICIPANTS: 76 men aged 60-86 yr | Testosterone  undecanoate 80 mg orally, twice daily.  T (chemiluminescent immunoassay) was 17.0 ± 4.4 nmol·L^-1^ for T group and 15.6 ± 4.5 nmol·L^-1^ for P group. At 12 mo declines of 1.7 ± 1.2 and 0.7 ± 1.1 nmol·L^-1^ were evident for T and P groups respectively. | Body composition | DXA | LBM increased in T (+1.54 kg) compared with  the placebo-treated group (-1.65 kg), *P* = 0.001). | - Immunoassay used to measure T - As part of inclusion criteria, men were required to have a free T index between 0.3-0.5 based on a single test. |
| Blackman *et al*. (11) (2002) | TYPE:  Randomized, double-blind placebo-controlled, parallel group  DURATION:  26 weeks  PARTICIPANTS: 38 men aged 65–88 yr with T levels <16.3 nmol·L^-1^ | Biweekly im injections of 100 mg testosterone.  T (radioimmunoassay - RIA) at baseline was 14.2 ± 0.7 nmol·L^-1^ for T group and 13.7 ± 0.8 nmol·L^-1^ for P group. Serum T increased in T group post-intervention (P<0.001), with no change for P group. | Body composition | DXA | No significant difference between T and placebo (*P* = 0.06). | - Immunoassay used to measure T. |
| Brill *et al*. (12) (2002) | TYPE:  Randomized cross-over  DURATION:  4-week interventions with 12-week washouts  PARTICIPANTS:  10 healthy older (60–78 yr) men with serum total T between 6.9–15.6 nmol·L^-1^. | 3 interventions:  - Transdermal T patch (5.0 mg·d^-1^)  - recombinant human GH (6.25 µg/·kg^-1^ sc daily)  - combined GH+T  T (solid-phase RIA) was increased by 62% above baseline. Baseline values not reported but were between 6.9 and 15.6 nmol·L^-1^ for eligibility. | Muscle gene expression | Bod-Pod for lean mass  Underwater weighing for fat mass | *Note*: Only T compared to baseline for purpose of this review. No significant difference between baseline and T for LBM. | - T measured with immunoassay - 3-month wash-out may have influenced results between two T interventions - A placebo patch and placebo injection condition were not used. |
| Ferrando *et al*. (13) (2002) | TYPE:  Double blind, randomized, placebo-controlled  DURATION:  26 weeks  PARTICIPANTS:  12 men >65 yr with total T <17 nmol·L^-1^ | Intramuscular injections weekly for 4 weeks then bi-weekly for 22 weeks to maintain T between 17-28 nmol·L^-1^.  T (by DPC, Los Angeles, CA) – dose was adjusted to maintain serum T between 17 and 28 nmol·L^-1^. | Histology parameters from muscle biopsies. | DXA | T increased LBM (+4.2 kg) significantly compared to P (‑2.0kg). | - Small numbers (T: *n* = 7, P: *n* = 5). - No specific T dose given so difficult to compare to other studies. |
| Kenny *et al.* (14) (2001) | TYPE:  Randomized, double-blind, placebo-controlled  DURATION:  52 weeks  PARTICIPANTS: 67 men aged 65–87 yr with bioavailable testosterone levels below 4.44 nmol·L^-1^ | Transdermal testosterone (two 2.5-mg patches  per day) or placebo patches for 1 year.  T pre-post (radioimmunoassay) was 13.7 ±5.6 to 20.3 ± 11.9 nmol·L^-1^ for T group and 14.2 ±7.2 to 16.6 ± 8.5 nmol·L^-1^ for P group. | Bone mineral density | DXA | No significant difference in LBM changes between T and placebo groups (*P =* 0.11) | - 35% attrition - T measured with immunoassay - Both groups also received 500 mg calcium and 400 IU Vit D supplements. |
| Wang *et al.* (15) (2000) | TYPE:  Randomized, multicenter (16 centers)  DURATION:  26 weeks  PARTICIPANTS: 167 men aged 19-68 (65% >65 yr) | 180 d treatment  with a 1% T gel preparation (50 or 100 mg·d^-1^, contained in 5 or 10 g gel, respectively) compared to a permeation-enhanced T  patch (5 mg·d^-1^). In the T gel groups, the T dose was adjusted up or down to 75  mg·d^-1^ (contained in 7.5 g gel) on day 90 if serum T was below or above the normal male range. No dose adjustment was  made in the T patch group.  Serum T was measured after extraction with ethyl acetate and hexane by a specific RIA. Baseline group means for serum T were 4.9 to 10.7 nmol·L^-1^ depending on hypogonadism category. | Sexual function | DXA | At 90 d of treatment, LBM increased more in the 100 mg·d^-1^ T gel group (+2.74 kg; *P =* 0.0002) than the 50 mg·d^-1^ T gel (+1.28 kg) and T patch groups (+1.20 kg). | - No control group - Multi-center trial, body composition measures not taken in 3 centers. |
| Clague *et al*. (16) (1999) | TYPE:  Double blind placebo-controlled  DURATION:  12 weeks  PARTICIPANTS:  14 men >60 yr (no age range provided)  with total T levels <14 nmol·L^-1^ | Random allocation to treatment with testosterone  enanthate 200 mg im placebo (castor oil) at 2 weekly intervals.  T (assay method not specified) increased from 11.3 ± 1.7 to 19.5 ± 4.8 nmol·L^-1^ in the T group; P group values were 11.6 ± 0.9 to 13.2 ± 2.0) nmol·L^-1^. | Muscle function | Bio-impedance meter | No significant difference between T and placebo groups. | - Small sample size (*n* = 7) per group. - Reliability of bioimpedance *vs* DXA. |
| Snyder *et al.* (17) (1999) | TYPE:  Double-blind, randomized, placebo controlled  DURATION:  156 weeks (3 years)  PARTICIPANTS: 108 healthy men > 65 yr whose serum testosterone concentration was 1 SD or more below the mean for normal young men (< 475 ng·dL^-1^/ 16.5 nmol·L^-1^). | Testosterone administered via scrotal patch. Placebo patches were identical in appearance to the testosterone patches. Each subject was asked to use a patch that delivered 6 mg·d^-1^ initially, to wear it at all times except when bathing, change the patch once a day, and to shave the scrotum once a week.  Mean serum T (method not specified) did not change in the P group; the T group increased from 12.7 ± 2.7 to 21.7 ± 8.6 nmol·L^-1^ by the 6^th^ month and remained at that level for the 36 mo of treatment. | Bone mineral density | DXA | Lean mass increased (+1.9 kg) in the T group which was significantly different (P < 0.001) from that (+0.2 kg) in the placebo group. | - Scrotal patch used for 36-month study, changed once daily, and removed when bathing. - Unclear how T was measured e.g., LC-MS / immunoassay. |
| Sih *et al*. (18) (1997) | TYPE: Randomized, double blinded,  placebo-controlled  DURATION:  52 weeks  PARTICIPANTS: 32 healthy men, 51–79 yr, serum bioavailable testosterone | Injections of testosterone cypionate:  200 mg every 2 weeks for 12 months  T (RIA) at baseline was 8.1 ± 0.7 nmol·L^-1^ in the P group and 10.2 ± 0.9 in the T group. 12 month values were 9.6 ± 0.8 and 12.8 ± 3.2 nmol·L^-1^. | Grip strength | Mid-arm circumfer-ence. | No change in mid-arm circumference | - Measurement of mid-arm circumference highly inaccurate compared to DXA. |
| Tenover *et al*. (19) (1992) | TYPE:  Randomized, double blinded,  placebo-controlled cross-over study  DURATION:  12 weeks  PARTICIPANTS: 13 men aged 57–76 yr, with T <13.9 nmol·L^-1^. | Testosterone enanthate im injections 100 mg weekly for 3 months  T (RIA) was 11.6 ± 0.4 nmol·L-^1^ (pooled) at baseline; post-intervention values were 19.7 ± 0.7 (T group) and 11.7 ± 0.6 nmol·L^-1^ (Placebo). | Body composition | Hydrodensitometry | 1·8 kg greater increase in fat-free mass with T compared to placebo. | - Washout period between conditions unclear - Less specific measurements of body mass (hydrodensi-tometry) - T measured via immunoassay - Small sample size (*n* = 13) - Some men given calcium carbonate if dietary recall showed intake was <1000 mg·d^-1^. |

DXA, dual-energy X-ray absorptiometry; ECLIA, electrochemoluminescence immunoassay; im, intramuscular; LBM, lean body mass; P, placebo; RIA, radioimmunoassay, T, testosterone.

**Table 2:** Studies investigating the effect of testosterone treatment and exercise on lean mass in healthy young and older men.

| **Young men (< 50 years)** | | | | | | | |
| --- | --- | --- | --- | --- | --- | --- | --- |
| **Source** | **Study  design** | **Testosterone intervention** | **Exercise**  **intervention** | **Primary outcome** | **Body composition method** | **Lean mass results** | **Limitations** |
| Pasiakos *et al.* (20) (2019) | TYPE: Double blind randomized placebo-controlled  DURATION:  14-d run-in, free-living, eucaloric diet phase; 28-d live-in, 55% exercise- and diet-induced energy deficit phase with (T or placebo)  PARTICIPANTS: 50 men aged 18–39 yr, with total testosterone  (10.4–34.7 nmol·L^-1^) | 200 mg testosterone enanthate  per week im injections  T (immunoassay) at baseline was 17 ± 5 and 15 ± 3 nmol·L^-1^ for T and P groups respectively. At the end of the treatment (Phase 2) T was 36.2 for the T group vs 12 nmol·L^-1^ for the P group. | Low-mod, and high intensity (40–85%  VO_2_peak aerobic-type exercise, including  treadmill and/or outdoor walking and/or running, elliptical, stationary  bike and weighted backpack (carrying 30% of body mass)  walking  Avg: 3.5 exercise sessions/day | Body comp. | DXA | T+Ex increased lean mass by 2.5 kg (P < 0.0001). No sig. change in placebo group. | - Severe exercise and diet induced-energy deficit representative of strenuous military training and combat operations - Not applicable to general population (avg of 3.5 exercise sessions per day) - No true control group |
| Giorgi *et al.* (21) (1999) | TYPE: Double blind randomized placebo-controlled  DURATION:  12 week administration + 12 week follow-up phase  PARTICIPANTS:  21 males (19-45 yr) with 2 years of weight training experience. No history of AAS use. | Intramuscular injection weekly of 3.5 mg·kg^-1^ bodyweight  Serum T was not reported. | Full body weight training 4 times·wk^-1^.  No specifics given as to %1RM for training. | Strength | Ultrasound scanning of rectus femoris and triceps brachii – lean. | The TE group increased rectus femoris circumference;  this increase was significantly greater than the placebo + exercise group. There were no differences recorded between the groups for triceps brachii thickness. | - Measurement of LBM conducted by ultrasound. - Measurement of body fat using skinfolds (calipers) - Both groups exercised so no true control group. |
| Bhasin *et al.* (22) (1996) | TYPE:  Double blind randomized placebo-controlled  4 groups:  - Testosterone + Exercise (T+Ex)  - Testosterone + No Exercise (T+NEx)  - Placebo + Exercise (P+Ex)  - Placebo + No Exercise (P+NEx)  DURATION:  10 weeks  PARTICIPANTS:  43 males (19-40 yr), experienced in weight-lifting. | Weekly 600 mg im injections  Serum T (immunoassay) pre and 10-week post values were:  - P+NEx 17.9 to 15.7 nmol·L^-1^  - T+NEx 17.4 to 98.1 nmol·L^-1^  - P+Ex 19.3 to 23.1 nmol·L^-1^  - T+Ex 15.0 to 112.6 nmol·L^-1^ | Resistance training 3x p/wk.  One session each at 90%, 80% and 70% 1RM per week.  Details of exact exercises not provided. | Muscle size | Muscle size:  MRI  Fat-free mass: underwater weighing | Muscle size: T groups  had significant increases in the cross-sectional areas of the triceps and the quadriceps. - -  - T+NEx > P+NEx  - T+Ex > P+Ex  Fat-free mass changes:  - P+NEx +0.8kg  - T+NEx +3.2 kg  - P+Ex +1.9 kg  - T+Ex +6.1 kg | - Potentially unable to be truly blinded due to such high T doses. |
| **Older men (> 50 years)** | | | | | | | |
| **Source** | **Study information** | **Testosterone intervention** | **Exercise**  **intervention** | **Primary outcome** | **Body composition method** | **Lean mass results** | **Limitations** |
| Barnouin *et al*. (23) (2021) | TYPE:  Double-blind randomized placebo-controlled  DURATION:  26 weeks  PARTICIPANTS:  83 men >65 yr with BMI >30 kg·m^-2^ and T <10.4 nmol·L^-1^ with mild-moderate frailty. | T gel 1.62% (40.5 mg) daily or placebo.  All participants were also prescribed a diet with an energy deficit of 500-750 kcal·day^-1^.  Serum T (liquid chromotography tandem mass spectrometry (LC-MS/MS)) at baseline was 7.6 ± 1.5 and 7.3 ± 2.2 nmol·L^-1^ in the P and T groups respectively. 6 month changes were +2.1 and +10.6 nmol·L^-1^ for P and T groups. | Combined aerobic and resistance training 3 x per week.  Aerobic: ~65% of peak HR, gradually  increased to 70-85%.  Resistance:  1-2 sets of 8-12 reps at 65% of 1RM, increased  to 2-3 sets at ~85% 1RM. | Physical perform-ance / function | DXA | LBM significantly decreased in P group compared to T  T: -1.2kg (1.8%) P: -2.4kg (3.5%) | - No true control group (all received the lifestyle intervention) - Average number of chronic diseases per participant was 4 and included heart failure, plus chronic lung and kidney disease. |
| Hildreth *et al.* (24) (2013) | TYPE:  Double-blind randomized placebo-controlled  DURATION:  52 weeks  PARTICIPANTS:  167 untrained community dwelling men ≥60 yr with total T between 6.9–12.1 nmol·L^-1^ | Placebo or transdermal T gel [2 doses targeting either a lower (400–550 ng·dL^-1^) or higher (600–1000 ng·dL^-1^) T range]  T (ELISA) at baseline averaged (pooled) 10.2 ± 1.3 nmol·L^-1^ in the P groups and 10.3 ± 1.5 in the T groups. 12 mo values were 10.0 ± 2.3 and 18.3 ± 9.9 nmol·L^-1^ for P and T groups respectively. | Full body progressive resistance training. Supervised 3 x p/wk. 50-70% 1RM. | Functional perfor-mance | DXA | P+NEx: +0.1kg  T+NEx: +1.0kg  P+Ex: +0.4kg  T+Ex: +2.1kg  T+Ex vs P+Ex, P < 0.05). | - Only 32% of men in the lower-range and 20% of men in the higher-range T group achieved target T levels at 52 weeks - Although designed to assess different T doses, all results were reported as high/low combined (‘any-T’). |
| Kvorning *et al.* (25) (2013) | TYPE:  Double blind randomized placebo-controlled  DURATION:  24 weeks (12 weeks strength training, followed by 12 weeks strength training +/- T)  PARTICIPANTS:  68 men 60–78 yr with low-normal bioavailable  testosterone levels (<7.3 nmol·L^-1^) and waist circ  >94 cm | 5g of gel containing 50 mg of testosterone  per day, for 12 weeks  T was measured using liquid chromatographys tandem mass spectrometry after ether extraction. A combination of strength training and T increased bioavailable T from 5.4 ± 0.6 to 10.7 ± 1.6 nmol·L^-1^. | The program was performed three times a week for  24 weeks and consisted of leg presses, knee extensions, leg  curls, chest presses, latissimus pull downs, back  extensions, and crunches | Mechanical muscle function | DXA | T+Ex increased LBM  approx. 2 kg /3%. No significant difference compared to strength training alone. | - Small numbers in exercise groups - T+Ex (*n* = 6), P+Ex (*n* = 8). |
| Katznelson *et al.* (26) (2006) | TYPE:  Double blind randomized placebo-controlled  DURATION:  12 weeks  PARTICIPANTS:  70 men aged 65–85 yr, free T <14.5 pg·mL^-1^ | 5 mg  transdermal system applied daily *vs* placebo system  Serum T (RIA) increased by a mean of 10.0 ± 1.9 (T+Ex), 6.6 ± 1.6 (T+Nex), 0.5 ± 0.6 (P+Ex) and 0.5 ± 0.6 (P+Nex) nmol·L^-1^ from baseline mean levels of 14.9, 13.6, 13.9 and 14.6 nmol·L^-1^ respectively. | Home-based resistance exercise program or no additional exercise | Quality of life | DXA | No effects of T or exercise on body composition parameters. | - Home-based exercise program (unsupervised) - No change in primary outcome either |
| Sullivan *et al.* (27) (2005) | TYPE:  Double blind randomized placebo-controlled  4 groups:  T or P combined with low- or high-level resistance training.  DURATION:  12 weeks  PARTICIPANTS:  71 men, 65-93 yr with recent decline in level of physical functioning. | Intramuscular injection of either testosterone enanthate (100 mg) or placebo.  T (method not specified) at baseline was 11.5 ± 6.0 and 10.2 ± 4.9 nmol·L^-1^ for placebo and T groups respectively (groups pooled across exercise intensity). Post-intervention, T groups (27.9 ± 9.8) were higher T than the P groups (10.5 ± 4.4) nmol·L^-1^. | 3 sessions per week.  Low resistance group = 20% 1RM  High resistance group = 80% 1RM. | Muscle strength | Mid-thigh fat-free muscle area: Computerised tomography. | Significant effect of T to increase mid-thigh cross sectional area in both T groups (T+high res: +8.6%, T+low res: +7.3%) compared to both placebo groups (P+high res: +1.2%, P+low res: +3.6%). | - No non-exercising group to assess effect of T alone. |

1RM, one repetition-maximum; DXA, dual-energy X-ray absorptiometry; im, intramuscular; LBM, lean body mass; P, placebo; T, testosterone.

**Table 3:** Studies investigating the effect of testosterone treatment on muscular strength and function in healthy middle-aged and older men.

| **Source** | **Study  design** | **Intervention** | **Primary outcome** | **Strength**  **assessment**  **technique** | **Strength results** | **Functional measures* / results** | **Limitations** |
| --- | --- | --- | --- | --- | --- | --- | --- |
| Borst *et al.* (1) (2014) | TYPE: Randomized, placebo-controlled  DURATION:  52 weeks  PARTICIPANTS:  30 men aged >60 yr, with a serum total T <10.4 nmol·L^-1^ | Testosterone: 125 mg·wk^-1^ im injection  Baseline T (Cobas electrochemoluminescence immunoassay) was 8.5 ± 2.5 nmol·L^-1^.  0-12mo change 7.9 (-0.9 to 16.8) nmol·L^-1^. | Muscle strength | 1RM leg press, knee flexion, knee extension, chest press, and triceps extension  Grip strength (dynamometer) | T sig. increased all strength measures compared to placebo.  - Leg press: 12.9 kg / +11.4%  - Knee extension: 6.0 kg / +8.1%  - Knee flexion: 5.4 kg +12.5%  - Chest press: 6.5 kg +14.5%  - Triceps extension: 5.3 kg / +9.5%  - Grip: 0.77 kg / +11.3% | No physical function tests measured. | - Men only excluded if received T within 4 weeks. - 33% attrition so small numbers in groups that finished the study (T: *n* = 7, P: *n* = 12) |
| Srinivas-Shankar *et al.* (4) (2010) | TYPE:  Randomized, double-blind, placebo-controlled, parallel group,  single-center  DURATION:  26 weeks  PARTICIPANTS: 274 community-dwelling intermediate-frail and frail elderly men >65 yr with total T ≤12 nmol·L^-1^. | Transdermal T (50 mg·d^-1^) or  placebo gel  T (chemiluminescent immunoassay): Baseline - T group 10.9 ± 3.1, P group 11.0 ±3.2 nmol·L^-1^.  6 mo – T group 18.4 ± 9.2, P group 10.7 ± 3.5 nmol·L^-1^ | Isometric knee extension peak torque  and isokinetic knee extension peak torque | Biodex knee extension on dominant leg | Isometric knee extension peak torque improved in the T group (*vs* placebo at 6 months),  adjusted difference was 8.6 N·m^-1^ (*P =* 0.02)  No difference in grip strength between groups. | Measures:  The aggregate locomotor function  test (ALF), physical performance test  (PPT), 6-min walk test (6MWT), and Tinetti gait and balance test.  Results: No significant differences between groups | - Single serum T measurement to determine eligibility - Diet and physical activities of the participants were not standardized - Time gap between randomization and baseline assessment - Twelve randomized men withdrew before baseline assessment and before they received the allocated treatment. |
| Sattler *et al*. (5) (2009) | TYPE:  Randomized, controlled, double-blinded  DURATION:  16 weeks  PARTICIPANTS: 39 participants aged 65-90 yr with T (150–550 ng·dL^-1^; 5.21–19.1 nmol·L^-1^) | 5 g or 10 g of 1% testosterone transdermal gel was applied each morning for 16 wk  T was  measured by liquid chromatography tandem mass spectrometry assay. 5g group baseline T was 13.4 ±3.7 nmol·L^-1^, 10g group was 12.1 ± 0.3 nmol·L^-1^. | Body composition | 1RM for bilateral leg press, leg extension, leg flexion, latissimus pull-down,  and chest press | No differences between groups.  (Results reported as composite 1RM). | Measure: Time to exhaustion for cycling VO_2_peak  Result: No differences between T and P groups.  Note: time to exhaustion was the only variable reported for this measure. No Wk 16 VO_2_peak values reported. | - No true control group (all received either T or rhGH), - All subjects were treated monthly from baseline to week 12 using a Leydig cell clamp with a long-acting GnRH agonist (leuprolide acetate depot, 7.5 mg im) - T measured using immunoassay |
| Emmelot-Vonk *et al.* (6) (2008) | TYPE:  Double-blind, randomized, placebo controlled  DURATION:  26 weeks  PARTICIPANTS: 237 healthy men 60-80 yr ; T<  13.7 nmol·L^-1^ | 2 capsules  of 40-mg testosterone undecenoate  twice per day with breakfast and dinner  (i.e. total dose of 160 mg·d^-1^  of testosterone undecenoate), or matching  placebo.  T (chemiluminescent enzyme immunoassay) in T group: 11.0 ± 1.9 nmol·L^-1^, P group 10.4 ±1.9. At 6mo, T was unchanged from baseline in the T group and increased slightly in the P group. Group difference at 6 mo was -3.2 nmol·L^-1^. | Functional mobility (Stanford Health Assessment Questionnaire,  timed get up and go test, isometric handgrip strength, isometric leg extensor  strength) | Isometric handgrip strength (hand dynamometer), isometric leg extensor  strength (measured using the MicroFET  hand-held dynamometer) | No difference between groups in any strength measures. | Measures: Functional mobility (Stanford Health Assessment Questionnaire,  timed get up and go test).  Results: No difference between groups in any physical function measures. | - Placebo group increased T levels more at 6 months than the T group - Oral capsules used - Isometric leg extensor strength measured using hand-held dynamometer |
| Storer *et al.* (7) (2008) | TYPE:  Double-blind, randomized study  DURATION:  20 weeks  PARTICIPANTS 44 community-dwelling healthy men aged 60-75 yr | Monthly treatment with a gonadotropin-releasing hormone agonist plus 25, 50, 125, or 300 mg·wk^-1^ of im injections of testosterone enanthate.  T: 25 mg (13 men), 50 mg (12 men), 125 mg (12 men), 300 mg (14 men), 600 mg (10 men).  Testosterone (immunoassay) pre-post values:  12.9 ± 4.4 to 6.1 ± 1.2; 11.4 ± 2.7 to 9.5 ± 0.6; 13.5 ± 4.5 to 29.6 ± 3.9 and 11.1 ± 3.8 to 61.9 ± 6.0 nmol·L^-1^ for the 25-, 50-, 125- and 300-mg·wk^-1^ dose groups respectively. | Skeletal muscle mass and strength | Maximal voluntary strength in the leg press exercise was measured as the one repetition maximum, 1rm, using a Keiser Seated Leg Press machine (Keiser Sport, Fresno, CA) with pneumatic resistance | Dose-dependent increases in leg press strength (*P* < 0.001) | Measures: Stair climbing, 6-m and 400-m walking speed, and a timed-up and-go (TUG).  Results: No difference between groups in any physical function measures. | - No control / placebo group - Data safety monitoring board discontinued the 600 mg study arm in older men because of a high frequency of serious adverse events. |
| [Giannoulis](javascript:;) *et al*. (8) (2006) | TYPE:  randomized, double-blind, placebo-controlled trial  DURATION:  26 weeks  PARTICIPANTS:  43 healthy, community-dwelling, older men (age, 65–80 yr). | T (5 mg) or placebo was given by transdermal patches  T (ADVIA Centaur analyzer) at baseline was 17 ± 1.2 nmol·L^-1^ for T group and 15 ±1.1 for P group; post intervention, T group 25 ± 3.3 nmol·L^-1^; no change P group | Body composition | Concentric knee flexion / extension using isokinetic dynamometer | No significant differences between T and placebo groups. | Measure: VO_2_peak  (also reported in Table 5).  Results: No significant differences between T and placebo groups. | - 30% developed skin irritations from T patches - Immunoassay used for measurement of T (instead of LC/MS) - VO_2_peak and strength assessments conducted during same testing session. |
| Page *et al*. (9) (2005) | TYPE:  Randomized, double blinded,  placebo-controlled  DURATION:  156 weeks (3 years)  PARTICIPANTS:  70 men aged 65-83 yr with T <350 ng·dL^-1^ (12.1 nmol·L^-1^) | 24 subjects received T only [T enanthate (TE);  200 mg im injection every 2 wk, plus a placebo pill orally (PO)  daily;  24 subjects received  placebo (sesame oil placebo), 1 mL im every 2 weeks, plus placebo pill PO  daily.  T (via fluoroimmunoassay): T group 9.9 ± 1.6, P group 10.5 ± 1.7 nmol·L^-1^ at baseline. Total T significantly increased for T but not P group over the treatment period. | Physical function | Handgrip (dynamometer)  Knee and ankle strength assessed using a Cybex  II isokinetic dynamometer | Sig. increase in right hand grip strength in T *vs* placebo (*P* = 0.01). No difference in left handgrip.  No differences between groups in lower extremity strength. | Measures:  - Rise from chair without using arms and then walk 50 ft, stepping over stacked boxes of various heights, walking through an open door, closing and opening the  door again, walking to a tiered platform to ascend and descend the stairs.  Results: Sig. difference between T and placebo (*P* < 0.01). T group decreased time to completion by 1.3 secs. Placebo increased time by 1.3 sec. | - Note: An additional 22 subjects received finasteride (F; which partially blocks the conversion of T to dihydrotestosterone). LBM results of T+F were similar to that of T alone. - 29% attrition rate - Men asked not to engage in regular exercise for 36 months |
| Wittert *et al.* (10) (2003) | TYPE: Randomized, double-blind, placebo-controlled study  DURATION:  52 weeks  PARTICIPANTS: 76 men aged 60–86 yr | Testosterone  undecanoate  80 mg orally, twice daily.  T (chemiluminescent immunoassay) was 17.0 ± 4.4 nmol·L^-1^ for T group and 15.6 ± 4.5 nmol·L^-1^ for P group. At 12 mo declines of 1.7 ± 1.2 and 0.7 ± 1.1 nmol·L^-1^ were evident for T and P groups respectively. | Body composition | Quadriceps and calf peak torque were measured  during concentric and eccentric maximal voluntary contraction  (MVC) using a KinCom isokinetic dynamometer  Grip strength (dynamometer) | No sig. differences in grip, quadriceps,  or calf strength between the T and placebo groups. | No physical function tests measured. | - Immunoassay used to measure T - As part of inclusion criteria, men were required to have a free T index between 0.3-0.5 based on a single test. |
| Blackman *et al*. (11) (2002) | TYPE:  Randomized, double-blind placebo-controlled, parallel group study  DURATION:  26 weeks  PARTICIPANTS: 38 men aged 65–88 yr with T levels <16.3 nmol·L^-1^ | Biweekly im injections of 100 mg testosterone  T (radioimmunoassay - RIA) at baseline was 14.2 ± 0.7 nmol·L^-1^ for T group and 13.7 ± 0.8 nmol·L^-1^ for P group. Serum T increased in T group post-intervention (P<0.001), with no change for P group. | Body composition | 1RM for bench press, upright row, arm curl, and arm extension, leg press and leg curl. | No difference between T and placebo groups (*P* = 0.86)  Total body strength was calculated as the sum of all six 1RM values. | No physical function tests measured. | - Immunoassay used to measure T. |
| Brill *et al*. (12) (2002) | TYPE:  Randomized cross-over study  DURATION:  4-week interventions with 12-week washouts  PARTICIPANTS:  10 healthy older (age, 60–78 yr) men with serum total T between 6.9–15.6 nmol·L^-1^ | 3 interventions:  - Transdermal T patch (5.0 mg·d^-1^)  - recombinant human GH (6.25 µg·kg^-1^ sc daily)  - combined GH+T  T (solid-phase RIA) was increased by 62% above baseline. Baseline values not reported but were between 6.9 and 15.6 nmol·L^-1^ for eligibility. | Muscle gene expression | Eccentric and concentric strength of the quadriceps femoris and biceps femoris muscle groups was assessed using isokinetic dynamometry | Note: Only T compared to baseline for purpose of this review.  No significant difference between baseline and following T treatment. | Measures:  - Functional ability was assessed via a four-flight stair climb and a 30-m walk. - Balance: timed one-legged stance  Results: No significant diff between baseline and following T treatment. | - T measured with immunoassay - 3-month wash-out may have influenced results between two T interventions - A placebo patch / placebo injection condition was not used. |
| Ferrando *et al*. (13) (2002) | TYPE:  Double blind, randomized, placebo-controlled  DURATION:  26 weeks  PARTICIPANTS:  12 men >65 yr with total T <17 nmol·L^-1^ | Intramuscular injections weekly for 4 weeks then bi-weekly for 22 weeks to maintain T between 17-28 nmol·L^-1^.  T (by DPC, Los Angeles, CA) – dose was adjusted to maintain serum T between 17 and 28 nmol·L^-1^. | Histology parameters from muscle biopsies | 1RM for bicep curl, tricep extension, leg extension, and leg curl | All 1RM strength measures increased significantly compared to placebo.  Bicep curl: +9.1kg  Tricep extension:  +10.4kg  Leg curl: +7.5kg  Leg extension: +15.3 | No physical function tests measured. | - Small numbers (T: *n* = 7, P: *n* = 5). - No specific T dose given so difficult to compare to other studies. |
| Kenny *et al.* (14) (2001) | TYPE:  Randomized, double-blind, placebo-controlled  DURATION:  52 weeks  PARTICIPANTS: 67 men aged 65– 87 yr with bioavailable testosterone levels below 4.44 nmol·L^-1^ | Transdermal testosterone (two 2.5-mg patches  per day) or placebo patches for 1 year.  T pre-post (radioimmunoassay) was 13.7 ±5.6 to 20.3 ± 11.9 nmol·L^-1^ for T group and 14.2 ±7.2 to 16.6 ± 8.5 nmol·L^-1^ for P group. | Bone mineral density | 1RM leg press | No differences between T and placebo groups (*P* = 0.66). | No physical function tests measured | - 35% attrition - T measured with immunoassay - Both groups also received 500 mg calcium and 400 IU Vit D supplements. |
| Wang *et al.* (15) (2000) | TYPE:  Randomized, multicenter (16 centers)  DURATION:  26 weeks  PARTICIPANTS: 167 men aged 19-68 (65% >65 yr) | 180 d treatment  with a 1% T gel preparation (50 or 100 mg·d^-1^, contained in 5 or 10 g gel, respectively) compared to a permeation-enhanced T  patch (5mg·d^-1^).  Serum T was measured after extraction with ethyl acetate and hexane by a specific RIA. Baseline group means for serum T were 4.9 to 10.7 nmol·L^-1^ depending on hypogonadism category. | Sexual function | 1RM technique in bench press and seated leg press  exercises. | Mean muscle  strength in the leg press exercise increased by 11 to 13 kg in all  treatment groups by 90 days and did not improve further at 180 days of treatment. Moderate increases were also observed in arm/chest  muscle strength. | No physical function tests measured | - No placebo group - Muscle strength was assessed in 167 of the 227 patients. Four of the 16 centers did not participate in the muscle strength testing because of lack of equipment. |
| Clague *et al*. (16) (1999) | TYPE:  Double blind placebo-controlled study  DURATION:  12 weeks  PARTICIPANTS:  14 men >60 yr (no age range provided) with total T levels <14 nmol·L^-1^ | Random allocation to treatment with testosterone  enanthate 200 mg im placebo (castor oil) at 2-weekly intervals  T (assay method not specified) increased from 11.3 ± 1.7 to 19.5 ± 4.8 nmol·L^-1^ in the T group; P group values were 11.6 ± 0.9 to 13.2 ± 2.0) nmol·L^-1^. | Muscle function | Isometric muscle strength of both knee  extensors and flexors was measured using a force transducer  fixed to a purpose-built chair.  Hand-grip – dynamometer. | No significant differences between T and placebo groups for any strength assessment. | Measures: Vertical step height  Results: No significant differences between T and placebo groups. | - Authors indicated they did not have statistical power for strength outcomes. |
| Snyder *et al.* (17) (1999) | TYPE:  Double-blind, randomized, placebo controlled  DURATION:  156 weeks (3 years)  PARTICIPANTS: 108 healthy men over 65 yr whose serum testosterone concentration was 1 SD or more below the mean for normal young men (<475 ng·dL^-1^/ 16.5 nmol·L^-1^). | Testosterone was administered by a scrotal patch); placebo patches were identical in appearance to the testosterone patches. Each subject was asked to use a patch that delivered 6 mg·d^-1^ initially, to wear it at all times except when bathing, change the patch once a day, and to shave the scrotum once a week.  Mean serum T (method not specified) did not change in the P group; the T group increased from 12.7 ± 2.7 to 21.7 ± 8.6 nmol·L^-1^ by the 6^th^ month and remained at that level for the 36 mo of treatment | Bone mineral density | Knee flexion and extension strength by Biodex dynamometer.  Hand grip (dynamometer) | No difference between T and placebo for knee strength or hand grip strength. | Measures: Physical function was assessed by walking (time and number of steps for a 25 ft walk at usual pace) and stair climbing (time for the subject to climb 12 stairs)  Results: Change in physical function tests did not differ between groups. | - Scrotal patch used for 36-month study, changed once daily and removed when bathing - Unclear how T was measured e.g. LC-MS / immunoassay. |
| Sih *et al*. (18) (1997) | TYPE: Randomized, double blinded,  placebo-controlled  DURATION:  52 weeks  PARTICIPANTS: 32 healthy men, 51–79 yr, serum bioavailable testosterone | Injections of testosterone cypionate, 200 mg every 2 weeks  T (RIA) at baseline was 8.1 ± 0.7 nmol·L^-1^ in the P group and 10.2 ± 0.9 in the T group. 12 month values were 9.6 ± 0.8 and 12.8 ± 3.2 nmol·L^-1^. | Grip strength | Hand grip strength  (dynamometer) | Testosterone significantly increased  grip strength for both left and right hands compared to placebo. | No physical function tests measured. | - Grip strength only. No lower body measures. |
| Urban *et al*. (28) (1995) | TYPE:  Preliminary study  DURATION:  4 weeks  PARTICIPANTS:  6 men aged 65-69 with total T <16.6 nmol·L^-1^ | 100 mg testosterone injections  T (RIA) values were used to adjust testosterone injection dosages to keep T concentrations within the normal range for younger men. | Skeletal muscle protein synthesis | Leg muscle strength measured with an isokinetic dynamometer. | Significant increase in strength for quadricep flexion and extension from baseline. | Measure: VO_2_peak  (also reported in Table 5).  Results: No significant difference from baseline. | - T measured via immunoassay. - No randomization or control group. - Small study (*n* = 6) - Short time frame (4 weeks) |
| Morley *et al*. (29) (1993) | TYPE:  Preliminary study  Alternate-case controlled trial  DURATION:  12 weeks  PARTICIPANTS: 14 males 69-89 yr | Testosterone enanthate (200 mg·mL^-1^) was administered intramuscularly to the treatment group every 2 weeks  T (RIA) mean value was not reported however 26/37 males had levels < 10.4 nmol·L^-1^. Mean baseline *bioavailable* T levels were 1.15 ± 0.14 and 1.28 ± 0.10 nmol·L^-1^ for control and T groups respectively; post-intervention: 1.35 ±0.35 and 11.2 ± 1.00 nmol·L^-1^ respectively. | Biochemical parameters | Hand grip strength  (dynamometer) | T significantly increased hand grip strength (*P* < 0.02) | Measure:  Timed single leg balance test  Result: No significant difference between T and placebo | - T measured via immunoassay - Small numbers (T: *n* = 8, P: *n* = 6). |
| Tenover *et al*. (19) (1992) | TYPE:  Randomized, double blinded,  placebo-controlled  DURATION:  12 weeks  PARTICIPANTS: 13 men aged 57–76 yr, with T <13.9 nmol·L^-1^ | Testosterone enanthate im injections 100 mg weekly for 3 months.  T (RIA) was 11.6 ± 0.4 nmol·L-^1^ (pooled) at baseline; post-intervention values were 19.7 ± 0.7 (T group) and 11.7 ± 0.6 nmol·L^-1^ (Placebo). | Body composition | Hand grip strength  (dynamometer) | No significant changes from baseline or between groups for handgrip strength | No physical function tests measured | - No washout period between conditions? - T measured via immunoassay - Grip strength only - Small sample (*n* = 13) - Some men given supplemental calcium carbonate if dietary recall showed intake was < 1000 mg·d-1. |

*For the purpose of this review, physical function excludes sexual / erectile function.

1RM, one repetition-maximum; im, intramuscular; LBM, lean body mass; P, placebo; T, testosterone.

**Table 4:** Studies investigating the effect of testosterone treatment and exercise on strength in healthy young and older men.

| **Young men (<50 years)** | | | | | | | | |
| --- | --- | --- | --- | --- | --- | --- | --- | --- |
| **Source** | **Study  design** | **Testosterone intervention** | **Exercise**  **intervention** | **Primary outcome** | **Strength**  **assessment**  **technique** | **Strength results** | **Functional measures* / results** | **Limitations** |
| Pasiakos *et al.* (20) (2019) | TYPE: Double blind randomized placebo-controlled  DURATION:  14-d run-in, free-living, eucaloric diet phase; 28-d live-in, 55% exercise- and diet-induced energy deficit phase with  Testosterone or placebo, and 14-d recovery, free-living, ad libitum diet phase.  PARTICIPANTS:  50 men aged 18–39 yr, with total testosterone 10.4–34.7 nmol·L^-1^ | 200 mg testosterone enanthate  per week im injections  T (immunoassay) at baseline was 17 ± 5 and 15 ± 3 nmol·L^-1^ for T and P groups respectively. At the end of the treatment (Phase 2) T was 36.2 for the T group vs 12 nmol·L^-1^ for the P group. | Low-mode and high-intensity (40–85% VO_2_peak) aerobic-type exercise, including  treadmill and/or outdoor walking and/or running, elliptical, stationary  bike and weighted backpack (carrying 30% of body mass)  walking. On average, 3.5 exercise sessions per day. | Body composition | Isometric and isokinetic knee extension tests (dynamo-meter) | Lower-body muscle function declined similarly for both groups during Phase 2, despite no loss of lean body mass in either group.  Type I slow-twitch myofiber cross-sectional area and percent myofiber  distribution increased, whereas type II fast-twitch myofiber cross sectional  area and percent distribution decreased during Phase 2 as compared to Phase 1, independent of treatment. | No physical function tests measured | - Severe exercise and diet induced-energy deficit representative of strenuous military training and combat operations. - Not applicable to general population - No true control group. |
| Giorgi *et al.* (21) (1999) | TYPE: Double blind randomized placebo-controlled  DURATION:  12 week administration + 12 week follow-up phase  PARTICIPANTS:  21 males (19-45 yr) with 2 years of weight training experience. No history of AAS use | Intramuscular injection weekly of 3.5 mg·kg^-1^ bodyweight.  Serum T was not reported. | - Full body weight training 4x·wk^-1^  - No specifics given as to %1RM for training | Strength | 1RM bench press | Testosterone: increased bench press 21 kg / 22%). Placebo increased 9 kg / 9%) | No physical function tests measured | - Measurement of LBM conducted by ultrasound - Both groups exercised so no true control group. |
| Bhasin *et al.* (22) (1996) | TYPE:  Double blind randomized placebo-controlled  4 groups:  - Testosterone + Exercise (T+Ex)  - Testosterone + No Exercise (T+NEx)  - Placebo + Exercise (P+Ex)  - Placebo + No Exercise (P+NEx)  DURATION:  10 weeks  PARTICIPANTS:  43 males (19-40 yr), experienced in weight-lifting. | Weekly 600 mg im injections.  Serum T (immunoassay) pre and 10-week post values were:  - P+NEx 17.9 to 15.7 nmol·L^-1^  - T+NEx 17.4 to 98.1 nmol·L^-1^  - P+Ex 19.3 to 23.1 nmol·L^-1^  - T+Ex 15.0 to 112.6 nmol·L^-1^ | Resistance training 3x p/wk: one session each at 90%, 80% and 70% 1RM per week.  Details of exact exercises not provided. | Muscle size | 1RM bench press and 1RM squat | Bench press:  - P+NEx: NC  - T+NEx: +10%  - P+Ex +11%  - T+Ex +38%  Squat:  - P+NEx: NC  - T+NEx: +19%  - P+Ex +21%  - T+Ex +22% | No physical function tests measured | - Potentially unable to be truly blinded due to such high T doses |
| **Older men (>50 years)** | | | | | | | | |
| **Source** | **Study  design** | **Testosterone intervention** | **Exercise**  **intervention** | **Primary outcome** | **Strength**  **assessment**  **technique** | **Strength results** | **Functional measures* / results** | **Limitations** |
| Barnouin *et al*. (23) (2021) | TYPE:  Double-blind randomized placebo-controlled  DURATION:  26 weeks  PARTICIPANTS:  83 men >65 yr with BMI > 30 kg·m^-2^ and T < 10.4 nmol·L^-1^ with mild-moderate frailty | T gel 1.62% (40.5 mg) daily or placebo  All participants also prescribed a diet with an energy deficit of 500-750 kcal·d^-1^.  Serum T (liquid chromotography tandem mass spectrometry (LC-MS/MS)) at baseline was 7.6 ± 1.5 and 7.3 ± 2.2 nmol·L^-1^ in the P and T groups respectively. 6 month changes were +2.1 and +10.6 nmol·L^-1^ for P and T groups. | Combined aerobic and resistance training 3 x per week.  Aerobic: ~65% peak HR, gradually  increased to 70-85%.  Resistance:  1-2 sets of 8-12 reps at 65% of 1RM, increased  to 2-3 sets at ~85% 1RM. | Physical performance test (PPT) | 1RM (biceps  curl, bench press, seated row, knee extension/  flexion, and leg press) | Reported as sum of 1RM: No significant differences between groups | Measures: PPT: 7 standardised  tasks requiring upper and lower body strength, flexibility, balance and coordination.  Note: also VO_2_peak as reported in Table 5  Results: PPT - No significant difference between T and P groups. | No true control group (all received the lifestyle intervention)   - Average number of chronic diseases per participant was 4 and included heart failure, plus chronic lung and kidney disease. |
| Hildreth *et al.* (24) (2013) | TYPE:  Double-blind randomized placebo-controlled  DURATION:  52 weeks  PARTICIPANTS:  167 untrained community dwelling men ≥60 yr with total T between 6.9–12.1 nmol·L^-1^ | Placebo or transdermal T gel [2 doses targeting either a lower (13.9–19.1 nmol·L^-1^) or higher (20.8–34.7 nmol·L^-1^) T range]  T (ELISA) at baseline averaged (pooled) 10.2 ± 1.3 nmol·L^-1^ in the P groups and 10.3 ± 1.5 in the T groups. 12 mo values were 10.0 ± 2.3 and 18.3 ± 9.9 nmol·L^-1^ for P and T groups respectively. | Full body progressive resistance training. Supervised 3 x p/wk. 50-70% 1RM. | Functional performance | 1RM measurements for bench press, incline press, overhead pull-down, and seated row, knee extension, knee flexion, and seated leg press | No difference between exercising groups for any measures.  For non-exercisers, T was associated with greater improvements in bench press, avg upper body strength and grip strength. | Measures: - Continuous physical function performance tests comprised of 15 everyday tasks requiring upper and lower body strength, flexibility, balance and coordination.  Results: No significant improvement with T in either group. | - Only 32% of men in the lower-range and 20% of men in the higher-range T group achieved target T levels at 52 weeks - Although designed to assess different T doses, all results reported as high/low combined (‘any-T’). |
| Kvorning *et al.* (25) (2013) | TYPE:  Double blind randomized placebo-controlled trial  DURATION:24 weeks (12 weeks strength training, followed by 2 weeks strength training +/- T)  PARTICIPANTS:  68 men 60–78 yr with low-normal bioavailable testosterone levels (<7.3 nmol·L^-1^) and waist circumference  >94 cm | 5 g of gel containing 50 mg·d^-1^, for 12 weeks.  T was measured using liquid chromatographys tandem mass spectrometry after ether extraction. A combination of strength training and T increased bioavailable T from 5.4 ± 0.6 to 10.7 ± 1.6 nmol·L^-1^. | The program was performed three times a week for 24 weeks and consisted of leg presses, knee extensions, leg curls, chest presses, latissimus pull downs, back extensions, and crunches. | Mechanical muscle function | Isometric and isokinetic knee extension tests  (dynamo-meter) | Combining testosterone therapy and strength training in a subgroup of men for an additional 12 weeks did not induce further increases in mechanical muscle function over strength training alone. | No physical function tests measured. | - Small numbers in exercise groups - T+Ex (*n* = 6), P+Ex (*n* = 8). |
| Katznelson *et al.* (26) (2006) | TYPE:  Double blind randomized placebo-controlled  DURATION:  12 weeks  PARTICIPANTS:  70 men aged 65–85 yr free T <14.5 pg·mL^-1^ | 5 mg·d^-1^  transdermal *vs* placebo system.  Serum T (RIA) increased by a mean of 10.0 ± 1.9 (T+Ex), 6.6 ± 1.6 (T+Nex), 0.5 ± 0.6 (P+Ex) and 0.5 ± 0.6 (P+Nex) nmol·L^-1^ from baseline mean levels of 14.9, 13.6, 13.9 and 14.6 nmol·L^-1^ respectively. | Home-based resistance exercise program or no additional exercise | Quality of life | Not measured | N/A | No physical function tests measured. | - Home-based exercise program (un-supervised) - No change in primary outcome (QoL) either |
| Sullivan *et al.* (27) (2005) | TYPE:  Double blind randomized placebo-controlled  4 groups:  T or P combined with low- or high-level resistance training  DURATION:  12 weeks  PARTICIPANTS:  71 men, 65-93 yr with a recent decline in level of physical functioning. | Intramuscular injection of testosterone enanthate (100 mg) or placebo.  T (method not specified) at baseline was 11.5 ± 6.0 and 10.2 ± 4.9 nmol·L^-1^ for placebo and T groups respectively (groups pooled across exercise intensity). Post-intervention, T groups (27.9 ± 9.8) were higher T than the P groups (10.5 ± 4.4) nmol·L^-1^. | 3 sessions per week.  Low resistance group = 20% 1RM  High resistance group = 80% 1RM. | Muscle strength | 1RM chest press and 1RM leg press | There was a trend toward greater strength improvement with T compared to placebo for both the arm and leg exercises, but neither of these differences reached statistical significance. | Measures: sit-to-stand, habitual gait speed test, stair climb,  Results: Neither intervention had a significant effect on functional performance | - No non-exercising group to assess effect of T alone. |

*For the purpose of this review, physical function excludes sexual / erectile function

1RM, one repetition-maximum; AAS, anabolic androgenic steroid; im, intramuscular; LBM, lean body mass; P, placebo; T, testosterone.

**Table 5:** Studies investigating the effect of testosterone treatment on VO_2_peak in healthy middle-aged and older men

| **Source** | **Study  design** | **Length** | **Participants** | **Intervention** | **Primary outcome** | **VO_2_peak results** | **Limitations** |
| --- | --- | --- | --- | --- | --- | --- | --- |
| Barnouin *et al*. (23) (2021) | Double-blind randomized placebo- controlled | 26 weeks | 83 men > 65 yrs with BMI > 30 kg·m^-2^, T < 10.4 nmol·L^-1^ and mild-moderate frailty | T gel 1.62% (40.5 mg·d^-1^) or placebo, in addition to exercise training and caloric restriction.  Serum T (liquid chromotography tandem mass spectrometry (LC-MS/MS)) at baseline was 7.6 ± 1.5 and 7.3 ± 2.2 nmol·L^-1^ in the P and T groups respectively. 6 month changes were +2.1 and +10.6 nmol·L^-1^ for P and T groups. | Physical function.  VO_2_peak measured via graded treadmill walking. | VO_2_peak increased significantly more in T (4.0 mL·kg^-1^·min^-1^ / 23%) than P (2.8 mL·kg^-1^·min^-1^ / 17%). | - No true control group (all received the lifestyle intervention) - Average number of chronic diseases per participant was 4 and included heart failure, plus chronic lung, and kidney disease - Low baseline VO_2_ values (between 5th – 10th percentile (30)) |
| Traustadottir *et al*. (31) (2018) | Double-blind, randomized, placebo-controlled, parallel-group  (Testosterone’s  Effects on Atherosclerosis Progression in Aging Men). | 156 wk (3 yr) | 129 men ≥60 yrs with total testosterone levels of 100 to 400 ng·dL^-1^ (3.5-13.9 nmol·L^-1^) | Randomization to 1% transdermal testosterone gel adjusted to achieve serum levels of 500 to 950 ng·dL^-1^ or placebo applied daily for 3 years.  T (immunoassay) at baseline was 11.0 ± 2.0 for P and 11.2 ± 2.0 nmol·L^-1^ for T group. Average of 6-, 18- and 36-mo levels was 20.0 ± 8.5; P group: 11.3 ± 3.4 nmol·L^-1^. | Change in VO_2_peak (cycle test) | VO_2_peak did not change in men treated with testosterone but fell in men receiving placebo (average 3-year decrease, 0.88 mL·kg^-1^·min^-1^;  *P* = 0.035).  Change in VO_2_peak between groups differed (average 3-year difference of 0.91 mL·kg^-1^·min^-1^; *P* = 0.008). | - The TEAAM trial started in 2004 when LCMS/MS was available in most laboratories - Participants with missing data at 6 and 18 months were proportionally similar. However, at 36 months, 28% of the placebo group had missing records compared with 12% missing records in men receiving testosterone. |
| Storer *et al*. (32) (2016) | Subgroup analysis of the Testosterone in Older Men with Mobility Limitations Trial | 26 weeks | 64 mobility-limited men deemed low cardiac risk, ≥65 yrs with low-serum total  testosterone (100–350 ng·dL^-1^ [3.5–12.1 nmol·L^-1^]. | Participants randomized to receive 100 mg testosterone gel or placebo gel  daily for 6 months.  T (immunoassay) baseline values were 9.1 ± 1.9 for T group and 8.4 ± 2.2 for P group. T group increase post intervention: 25 ± 15 nmol·L^-1^. P group increased by 2.2 ± 6.0 nmol·L^-1^. | VO_2_peak from a symptom-limited cycle exercise test | VO_2_peak increased by 0.83 (2.4) mL·kg^-1^·min^-1^ in testosterone but decreased by  0.89 (2.5) mL·kg^-1^·min^-1^ in placebo (*P* = 0.035); between group difference in change in VO_2_peak was  significant (*P =* 0.006). | - 6-month reduction in placebo was greater than the expected 0.4- mL·kg-1·min-1·yr-1 rate of decline in the general population. I.e., difference between treatment groups resulted from decline in placebo group as opposed to improvement in T group. - Immunoassay used for measurement of T (instead of LC/MS). |
| Nair *et al*. (33) (2006) | Placebo-controlled, randomized, double-blind | 104 weeks | 58 men  ≥ 60 yrs of age  Eligibility  criteria: T ≤ 103 ng·dL^-1^  (≤ 3.6 nmol·L^-1^) and free from co-existing illnesses or conditions that may have affected outcomes. | Transdermal testosterone  patch (5 mg per day; or placebo)  T (competitive chemiluminescence immunoassay) at baseline: P group: 13.8 nmol·L^-1^, T group 12.4 nmol·L^-1^. The T but not P group had a significant increase in total T (values not given). | VO_2_peak was measured  during graded-intensity treadmill-walking. | There was no significant difference between T and placebo groups: median difference 0.48 mL·kg^-1^·min^-1^, (*P =* 0.83). | - Very low baseline T level (3.6 nmol·L-1) - Immunoassay used for measurement of T (instead of LC/MS) - Baseline VO_2_peak was high = 40.7 and 40.4 mL·kg-1·min-1 (in the T and placebo groups respectively) which places these men at the 90th percentile for males aged between 60-69 yrs (30). |
| [Giannoulis](javascript:;) *et al*. (8) (2006) | Randomized, double-blind, placebo-controlled | 26 weeks | 43 healthy, community-dwelling, older men (age, 65– 80 yrs) | Testosterone (5 mg) or placebo was given by transdermal patches.  T (ADVIA Centaur analyzer) at baseline was 17 ± 1.2 nmol·L^-1^ for T group and 15 ±1.1 for P group; post intervention, T group 25 ± 3.3 nmol·L^-1^; no change P group. | Body composition; however, VO_2_peak also measured using maximal cycle test. | No significant difference between T and placebo groups | - 30% developed skin irritations from T patches - Immunoassay used for measurement of T (instead of LC/MS) - VO_2_peak and strength assessments conducted during same testing session. |
| Blackman *et al*. (11) (2002) | Randomized, double-blind placebo-controlled, parallel group | 26 weeks | 38 men aged 65–88 yrs with T levels < 16.3 nmol·L^-1^ | Biweekly im injections of 100 mg testosterone.  T (radioimmunoassay - RIA) at baseline was 14.2 ± 0.7 nmol·L^-1^ for T group and 13.7 ± 0.8 nmol·L^-1^ for P group. Serum T increased in T group post-intervention (P<0.001), with no change for P group. | Body composition; however VO_2_peak was measured using a symptom-limited graded treadmill test. | P group declined significantly more than T group (T: ‑0.4 *vs* P: ‑1.2 mL·kg^-1^·min^-1^). | - Immunoassay used for measurement of T (instead of LC/MS). |
| Urban *et al*. (28) (1995) | Preliminary study | 4 weeks | 6 men aged 65-69 with total T <16.6 nmol·L^-1^ | 100 mg testosterone injections.  T (RIA) values were used to adjust testosterone injection dosages to keep T concentrations within the normal range for younger men. | Skeletal muscle protein synthesis, however, VO_2_peak also measured using maximal cycle test. | No significant difference between T and baseline. | - T measured via immunoassay - No randomization or control group - Small study (n = 6) - Short time frame (4 weeks) |

BMI, body mass index; im, intramuscular; P, placebo; T; testosterone.

**REFERENCES**

1. Borst SE, Yarrow JF, Conover CF, Nseyo U, Meuleman JR, Lipinska JA, et al. Musculoskeletal and prostate effects of combined testosterone and finasteride administration in older hypogonadal men: a randomized, controlled trial. Am J Physiol Endocrinol Metab. 2014;306(4):E433-E42.

2. Behre HM, Tammela TL, Arver S, Tolra JR, Bonifacio V, Lamche M, et al. A randomized, double-blind, placebo-controlled trial of testosterone gel on body composition and health-related quality-of-life in men with hypogonadal to low-normal levels of serum testosterone and symptoms of androgen deficiency over 6 months with 12 months open-label follow-up. Aging Male. 2012;15(4):198-207.

3. Frederiksen L, Hojlund K, Hougaard DM, Brixen K, Andersen M. Testosterone therapy increased muscle mass and lipid oxidation in aging men. Age (Dordr). 2012;34(1):145-56.

4. Srinivas-Shankar U, Roberts SA, Connolly MJ, O'Connell MD, Adams JE, Oldham JA, et al. Effects of testosterone on muscle strength, physical function, body composition, and quality of life in intermediate-frail and frail elderly men: a randomized, double-blind, placebo-controlled study. J Clin Endocrinol Metab. 2010;95(2):639-50.

5. Sattler FR, Castaneda-Sceppa C, Binder EF, Schroeder ET, Wang Y, Bhasin S, et al. Testosterone and growth hormone improve body composition and muscle performance in older men. J Clin Endocrinol Metab. 2009;94(6):1991-2001.

6. Emmelot-Vonk MH, Verhaar HJ, Nakhai Pour HR, Aleman A, Lock TM, Bosch JL, et al. Effect of testosterone supplementation on functional mobility, cognition, and other parameters in older men: a randomized controlled trial. JAMA. 2008;299(1):39-52.

7. Storer TW, Woodhouse L, Magliano L, Singh AB, Dzekov C, Dzekov J, et al. Changes in muscle mass, muscle strength, and power but not physical function are related to testosterone dose in healthy older men. J Am Geriatr Soc. 2008;56(11):1991-9.

8. Giannoulis MG, Sonksen PH, Umpleby M, Breen L, Pentecost C, Whyte M, et al. The effects of growth hormone and/or testosterone in healthy elderly men: a randomized controlled trial. J Clin Endocrinol Metab. 2006;91(2):477-84.

9. Page ST, Amory JK, Bowman FD, Anawalt BD, Matsumoto AM, Bremner WJ, et al. Exogenous testosterone (T) alone or with finasteride increases physical performance, grip strength, and lean body mass in older men with low serum T. J Clin Endocrinol Metab. 2005;90(3):1502-10.

10. Wittert GA, Chapman IM, Haren MT, Mackintosh S, Coates P, Morley JE. Oral testosterone supplementation increases muscle and decreases fat mass in healthy elderly males with low-normal gonadal status. J Gerontol A Biol Sci Med Sci. 2003;58(7):618-25.

11. Blackman MR, Sorkin JD, Munzer T, Bellantoni MF, Busby-Whitehead J, Stevens TE, et al. Growth hormone and sex steroid administration in healthy aged women and men: a randomized controlled trial. JAMA. 2002;288(18):2282-92.

12. Brill KT, Weltman AL, Gentili A, Patrie JT, Fryburg DA, Hanks JB, et al. Single and combined effects of growth hormone and testosterone administration on measures of body composition, physical performance, mood, sexual function, bone turnover, and muscle gene expression in healthy older men. J Clin Endocrinol Metab. 2002;87(12):5649-57.

13. Ferrando AA, Sheffield-Moore M, Yeckel CW, Gilkison C, Jiang J, Achacosa A, et al. Testosterone administration to older men improves muscle function: molecular and physiological mechanisms. Am J Physiol Endocrinol Metab. 2002;282(3):E601-7.

14. Kenny AM, Prestwood KM, Gruman CA, Marcello KM, Raisz LG. Effects of transdermal testosterone on bone and muscle in older men with low bioavailable testosterone levels. J Gerontol A Biol Sci Med Sci. 2001;56(5):M266-72.

15. Wang C, Swerdloff RS, Iranmanesh A, Dobs A, Snyder PJ, Cunningham G, et al. Transdermal testosterone gel improves sexual function, mood, muscle strength, and body composition parameters in hypogonadal men. J Clin Endocrinol Metab. 2000;85(8):2839-53.

16. Clague JE, Wu FC, Horan MA. Difficulties in measuring the effect of testosterone replacement therapy on muscle function in older men. Int J Androl. 1999;22(4):261-5.

17. Snyder PJ, Peachey H, Hannoush P, Berlin JA, Loh L, Lenrow DA, et al. Effect of testosterone treatment on body composition and muscle strength in men over 65 years of age. J Clin Endocrinol Metab. 1999;84(8):2647-53.

18. Sih R, Morley JE, Kaiser FE, Perry HM, 3rd, Patrick P, Ross C. Testosterone replacement in older hypogonadal men: a 12-month randomized controlled trial. J Clin Endocrinol Metab. 1997;82(6):1661-7.

19. Tenover JS. Effects of testosterone supplementation in the aging male. J Clin Endocrinol Metab. 1992;75(4):1092-8.

20. Pasiakos SM, Berryman CE, Karl JP, Lieberman HR, Orr JS, Margolis LM, et al. Effects of testosterone supplementation on body composition and lower-body muscle function during severe exercise- and diet-induced energy deficit: A proof-of-concept, single centre, randomised, double-blind, controlled trial. EBioMedicine. 2019;46:411-22.

21. Giorgi A, Weatherby RP, Murphy PW. Muscular strength, body composition and health responses to the use of testosterone enanthate: a double blind study. J Sci Med Sport. 1999;2(4):341-55.

22. Bhasin S, Storer TW, Berman N, Callegari C, Clevenger B, Phillips J, et al. The effects of supraphysiologic doses of testosterone on muscle size and strength in normal men. N Engl J Med. 1996;335(1):1-7.

23. Barnouin Y, Armamento-Villareal R, Celli A, Jiang B, Paudyal A, Nambi V, et al. Testosterone replacement therapy added to intensive lifestyle intervention in older men with obesity and hypogonadism. J Clin Endocrinol Metab. 2021;106(3):e1096-e110.

24. Hildreth KL, Barry DW, Moreau KL, Vande Griend J, Meacham RB, Nakamura T, et al. Effects of testosterone and progressive resistance exercise in healthy, highly functioning older men with low-normal testosterone levels. J Clin Endocrinol Metab. 2013;98(5):1891-900.

25. Kvorning T, Christensen LL, Madsen K, Nielsen JL, Gejl KD, Brixen K, et al. Mechanical muscle function and lean body mass during supervised strength training and testosterone therapy in aging men with low-normal testosterone levels. J Am Geriatr Soc. 2013;61(6):957-62.

26. Katznelson L, Robinson MW, Coyle CL, Lee H, Farrell CE. Effects of modest testosterone supplementation and exercise for 12 weeks on body composition and quality of life in elderly men. Eur J Endocrinol. 2006;155(6):867-75.

27. Sullivan DH, Roberson PK, Johnson LE, Bishara O, Evans WJ, Smith ES, et al. Effects of muscle strength training and testosterone in frail elderly males. Med Sci Sports Exerc. 2005;37(10):1664-72.

28. Urban RJ, Bodenburg YH, Gilkison C, Foxworth J, Coggan AR, Wolfe RR, et al. Testosterone administration to elderly men increases skeletal muscle strength and protein synthesis. Am J Physiol. 1995;269(5 Pt 1):E820-6.

29. Morley JE, Perry HM, 3rd, Kaiser FE, Kraenzle D, Jensen J, Houston K, et al. Effects of testosterone replacement therapy in old hypogonadal males: a preliminary study. J Am Geriatr Soc. 1993;41(2):149-52.

30. Kaminsky LA, Arena R, Myers J. Reference standards for cardiorespiratory fitness measured with cardiopulmonary exercise testing: Data from the fitness registry and the importance of exercise national database. Mayo Clin Proc. 2015;90(11):1515-23.

31. Traustadottir T, Harman SM, Tsitouras P, Pencina KM, Li Z, Travison TG, et al. Long-term testosterone supplementation in older men attenuates age-related decline in aerobic capacity. J Clin Endocrinol Metab. 2018;103(8):2861-69.

32. Storer TW, Bhasin S, Travison TG, Pencina K, Miciek R, McKinnon J, et al. Testosterone attenuates age-related fall in aerobic function in mobility limited older men with low testosterone. J Clin Endocrinol Metab. 2016;101(6):2562-9.

33. Nair KS, Rizza RA, O'Brien P, Dhatariya K, Short KR, Nehra A, et al. DHEA in elderly women and DHEA or testosterone in elderly men. N Engl J Med. 2006;355(16):1647-59.
